# Comparative genomics and phylogenetic relationships of two endemic and endangered species (*Handeliodendron bodinieri* and *Eurycorymbus cavaleriei*) of two monotypic genera within Sapindales

**DOI:** 10.1186/s12864-021-08259-w

**Published:** 2022-01-06

**Authors:** Jiaxin Yang, Guoxiong Hu, Guangwan Hu

**Affiliations:** 1 Core Botanical Gardens/Wuhan Botanical Garden, Chinese Academy of Sciences, Wuhan, 430074 China; 2 Sino-Africa Joint Research Center, Chinese Academy of Sciences, Wuhan, 430074 China; 3 University of Chinese Academy of Sciences, Beijing, 100049 China; 4 College of Life Sciences, Guizhou University, Guiyang, 550025 Guizhou China

**Keywords:** Endangered, Monotypic genus, Endemic, *Handeliodendron*, *Eurycorymbus*, Chloroplast genome, Phylogenetic analysis, Sapindaceae

## Abstract

**Background:**

*Handeliodendron* Rehder and *Eurycorymbus* Hand.-Mazz. are the monotypic genera in the Sapindaceae family. The phylogenetic relationship of these endangered species *Handeliodendron bodinieri* (Lévl.) Rehd. and *Eurycorymbus cavaleriei* (Lévl.) Rehd. et Hand.-Mazz. with other members of Sapindaceae s.l. is not well resolved. A previous study concluded that the genus *Aesculus* might be paraphyletic because *Handeliodendron* was nested within it based on small DNA fragments. Thus, their chloroplast genomic information and comparative genomic analysis with other Sapindaceae species are necessary and crucial to understand the circumscription and plastome evolution of this family.

**Results:**

The chloroplast genome sizes of *Handeliodendron bodinieri* and *Eurycorymbus cavaleriei* are 151,271 and 158,690 bp, respectively. Results showed that a total of 114 unique genes were annotated in *H. bodinieri* and *E. cavaleriei*, and the *ycf1* gene contained abundant SSRs in both genomes. Comparative analysis revealed that gene content, PCGs, and total GC content were remarkably similar or identical within 13 genera from Sapindaceae, and the chloroplast genome size of four genera was generally smaller within the family, including *Acer*, *Dipteronia*, *Aesculus*, and *Handeliodendron*. IR boundaries of the *H. bodinieri* showed a significant contraction, whereas it presented a notable expansion in *E. cavaleriei* cp genome. *Ycf1*, *ndhC-trnV-UAC*, and *rpl32-trnL-UAG-ccsA* were remarkably divergent regions in the Sapindaceae species. Analysis of selection pressure showed that there are a few positively selected genes. Phylogenetic analysis based on different datasets, including whole chloroplast genome sequences, coding sequences, large single-copy, small single-copy, and inverted repeat regions, consistently demonstrated that *H. bodinieri* was sister to the clade consisting of *Aesculus chinensis* and *A. wangii* and strongly support *Eurycorymbus cavaleriei* as sister to *Dodonaea viscosa*.

**Conclusion:**

This study revealed that the cp genome size of the Hippocastanoideae was generally smaller compared to the other subfamilies within Sapindaceae, and three highly divergent regions could be used as the specific DNA barcodes within Sapindaceae. Phylogenetic results strongly support that the subdivision of four subfamilies within Sapindaceae, and *Handeliodendron* is not nested within the genus *Aesculus*.

**Supplementary Information:**

The online version contains supplementary material available at 10.1186/s12864-021-08259-w.

## Introduction

*Handeliodendron bodinieri* (Lévl.) Rehd. and *Eurycorymbus cavaleriei* (Lévl.) Rehd. et Hand.-Mazz. (Sapindaceae) are deciduous woody plants endemic to China, and belong to the two monotypic genera *Handeliodendron* and *Eurycorymbus*, respectively [1]. Previous studies revealed that *H. bodinieri* and *E. cavaleriei* are economically important plants. Their seed kernel have been used as raw materials for producing biodiesel, sources of protein and edible oil and are of high medicinal and nutritional values [2, 3]. *H. bodinieri* species are mainly distributed in the south Guizhou province and the northwest of Guangxi Zhuang Autonomous Region [1], and a small population is found in Yunan province, China [4]. *E. cavaleriei* are distributed in Fujian, Guangdong, Guangxi, Guizhou, Hunan, Jiangxi, Sichuan, Taiwan, and Yunnan provinces [5]. The two species are categorized as endangered according to Information System of Chinese Rare and Endangered Plants (ISCREP) (http://www.iplant.cn/rep/). Significantly, *H. bodinieri* has been listed among the level-I state-protected wild plants, whereas *E. cavaleriei* has been classified as the level-II state-protected wild plants in the list. In addition, *E. cavaleriei* was designated as Near Threatened (NT) by the International Union for Conservation of Nature (IUCN) Red List (https://www.iucnredlist.org/species/31353/9628640).

Historically, there were considerable controversies about the circumscription of Sapindaceae, predominantly the traditionally defined Aceraceae and Hippocastanaceae should be incorporated into Sapindaceae or separated. The concept of Sapindaceae s. str. Was first proposed by Jussieu in 1789, that supported that the family is different from Aceraceae. After this, the works of Radlkofer maintained the family distinct from Aceraceae and Hippocastanaceae, and proposed the first worldwide system of classification for Sapindaceae [6, 7]. In this classification system of Radlkofer, Sapindaceae was divided into two subfamilies Eusapindaceae and Dyssapindaceae. The Eusapindaceae consists of nine tribes, including Paullinieae, Thouinieae, Sapindeae, Aphanieae, Lepisantheae, Melicocceae, Nephelieae, and Cupanieae, whereas the Dyssapindaceae contains five tribes (including Koelreuterieae, Cossignieae, Doratoxyleae, and Harpullieae). Muller and Leenhouts [8] proposed the revised classification of Sapindaceae based upon the macromorphology and pollen morphology, in which the tribe Aphanieae was incorporated into Lepisantheae. They considered Aceraceae and Hippocastanaceae as related to the Sapindaceae basing on pollen character, thus suggested that they be retained as tribes within the Sapindaceae. However, most classification systems [9–11] maintained Aceraceae and Hippocastanaceae as distinct families. On the basis of phytochemistry, Umadevi and Daniel [12] proposed a system of classification for Sapindaceae. They divided the family into four subfamilies: Aceroideae (including all members of the former Aceraceae), Sapindoideae (including the former Hippocastanaceae as a tribe), Dodoneoideae (*Dodonaea*), and Koelreuterioideae (all the other tribes of Dodoneoideae of Radlkofer). Judd [13] agreed with the broader concept of Sapindaceae and supported the inclusion of both families within Sapindaceae. The system of Thorne [14] divided the family into five subfamilies: Dodonaeoideae, Koelreuterioideae, Sapindoieae, Hippocastanoideae (*Aesculus* and *Billia*), and Aceroideae (*Acer* and *Dipteronia*). Based on plastid genes *matK* and *rbcL*, Harrington et al. [15] conducted the phylogenetic analysis in Sapindaceae, and supported the recognition of a broadly defined Sapindaceae incorporating Aceraceae and Hippocastanaceae. They further proposed that the subdivision of four subfamilies within Sapindaceae: Xanthoceroideae (including the single genus *Xanthoceras*), Hippocastanoideae, Dodonaeoideae, and Sapindoideae. The work of Harrington et al. [15] has been adopted by Thorne et al. [16]. By increasing taxon sampling and expanding DNA data, Buerki et al. [17] revealed the relationships at subfamilial and tribal levels in Sapindaceae. They supported the merging of Aceraceae and Hippocastanaceae into Sapindaceae, and recognized four subfamilies within Sapindaceae (Xanthoceroideae, Hippocastanoideae, Dodonaeoideae, and Sapindoideae). Nevertheless, based on molecular sequence data, morphology and biogeography, Buerki et al. [18] resurrected the traditional families Aceraceae and Hippocastanaceae and further proposed a new family, Xanthoceraceae (including the single genus *Xanthoceras*). The concept of a broadly defined Sapindaceae that includes Aceraceae, Hippocastanaceae, and *Xanthoceras*, was adopted by the newly published Angiosperm Phylogeny Group (APG) [19].

For the *Handeliodendron bodinieri,* Rehder [20] proposed the new genus *Handeliodendron*, and it resembles the Hippocastanaceae in opposite and digitately 5-foliolate leaves. However, other characters showed that it is a closer affinity with the Sapindaece and is best placed in the tribe Harpullieae [20]. The work of Muller and Leenhouts [8] considered that *Delavaya* and *Handeliodendron* are the intermediate between Harpullieae and Hippocastanaceae. Judd [13] supported that *Handeliodendron* should be classified together with members of the Hippocastanaceae. The molecular phylogenetic analysis of Sapindaceae demonstrated that *Handeliodendron* should be transferred from Harpullieae to tribe Hippocastaneae (subfamily Hippocastanoideae) containing *Aesculus* and *Billia*, and strongly supported *H. bodinieri* was sister to the *Aesculus* plus *Billia* [15]. According to *Flora of China*, Xia et al. [21] maintained the Hippocastanaceae as a distinct family, comprised of *Handeliodendron*, *Aesculus*, and *Billia*. Buerki et al. [17] supported that *Handeliodendron*, *Aesculus*, and *Billia* were members of the *Aesculus* group in the subfamily Hippocastanoideae, but their work lacked samples of *Handeliodendron* and *Billia*. By increasing samples and DNA markers, Buerki et al. [18] concluded the genus *Aesculus* might be paraphyletic because the *Handeliodendron* and *Billia* were nested within it, but the relationship was only weakly supported (for *Handeliodendron*: BS = 68; for *Billia*: BS = 76). For the genus *Eurycorymbus*, it was a component of the Harpullieae in the subfamily Dodonaeoideae [6, 8]. Molecular analyses indicated that *Eurycorymbus* belongs to a member of the *Dodoneae* group in the Dodonaeoideae, the results strongly supported *E. cavaleriei* was sister to *Euphorianthus longifolius* [17, 18].

Despite the systematic position of *Handeliodendron bodinieri* and *Eurycorymbus cavaleriei* tending to show more stability within the Sapindaceae, previous studies were mainly based on the morphology and/or limited DNA regions. At present, the next-generation sequencing (NGS) technologies and bioinformatics tools bring great convenience for acquiring and analyzing genome-scale data, while obtaining genome-scale nuclear data remains significantly difficult in terms of expense. On the contrary, the plastid genome has become extremely easy to obtain because of its highly conserved nature and much smaller size. Meanwhile, an increasing number of the chloroplast genomes have been widely applied to solve phylogenetic relationships at different taxonomical levels within angiosperms [22–29]. Besides, recent studies have demonstrated chloroplast genome can serve as super barcodes for species and taxonomic groups [23, 30–33]. There are some cp genomes in Sapindaceae s.l. species have been reported and deposited in GenBank database, like *Acer* [34], *Dodonaea* [35], *Aesculus* [36–39], *Dimocarpus* [40], most of which were the genus *Acer* [41–46]. We also noticed that previous studies have revealed the complete cp genomes of *H. bodinieri* and *E. cavaleriei* [47–49], but focus mainly on the size and gene contents of the cp genome, lacking detailed comparative genomic and comprehensive phylogenetic analysis. Moreover, a comprehensive genomics analysis is still a useful framework for understanding plastome evolution and phylogenetic relationships within Sapindaceae. Getorganelle [50] was used to accurately assemble the cp genome of *H. bodinieri* and *E. cavaleriei*, which underpins the further study. Due to insufficient molecular data, the result of Tian, et al. [49] showed *H. bodinieri* is closely related to the genus *Mangifera*. However, according to the work of Harrington et al. [15], *H. bodinieri* was closely related to *Aesculus* plus *Billia*. Therefore, it is necessary to perform a comprehensive cp genomic comparison and phylogenetic analysis in the Sapindaceae.

In the current study, we sequenced and assembled the complete chloroplast genomes of *Handeliodendron bodinieri* and *Eurycorymbus cavaleriei*. The main objectives of this study were to 1) compare and analyze the gene organizations of *H. bodinieri* and *E. cavaleriei* cp genomes; 2) reveal the cp genome structural and size variation in Sapindaceae; 3) explore the highly divergent regions of the cp genomes from Sapindaceae species; 4) reconstruct the phylogeny of *H. bodinieri* and *E. cavaleriei* within Sapindales at the cp genome level.

## Materials and methods

### Plant material and DNA extraction

Fresh leaves of cultivated plants *Handeliodendron bodinieri* (voucher Number: HXZ-Z-0001) and *Eurycorymbus cavaleriei* (voucher Number: Hu-Z-0001) were collected from Guizhou Botanical Garden and Pingtang county of Guizhou, China, respectively. These sample collections were approved by the Guizhou Academy of Sciences and Guizhou Forestry Bureau, Guizhou province, China. The voucher specimens were deposited in the Herbarium of Nature Museum, Guizhou University (GACP). Total genomic DNA of *E. cavaleriei* was extracted following a modified cetyltrimethylammonium bromide (CTAB) approach [51], while that of *H. bodinieri* could not be obtained by the same method. Total genomic DNA of *H. bodinieri* was eventually extracted using a CTAB approach as improved by Tong [52] .

### Chloroplast genome sequencing, assembly and annotation

The genome was sequenced using the Illumina Hiseq 2500 platform at Wuhan BGI Technology Service Co., Ltd. (Wuhan, China). Complete chloroplast genomes of *Handeliodendron bodinieri* and *Eurycorymbus cavaleriei* were assembled from raw reads using GetOrganelle v1.7.1 with default parameters [50], which will automatically call the SPAdes [53], Bowtie2 [54] and BLAST [55]. The assembly workflow includes five key steps: 1). Mapping reads to seed and assembling seed-mapped reads for parameter estimation; 2). Recruiting more target-associated reads through extending iterations; 3). Conducting de novo assembly; 4). Roughly filtering for target-like contigs; 5). Identifying target contigs and exporting all configurations [50]. The final assembly results were checked using Bandage [56].

Complete chloroplast genomes were annotated using the PGA (Plastid Genome Annotator) software [57], with *Litchi chinensis* (NC_035238) as main reference. Subsequently, Geneious 11.0.4 [58] was used to manually adjusted start and stop codons based on multiple cp genomes of Sapindaceae species. The structural features of cp genome maps were drawn online using OGDRAW [59]. The final cp genomes of *Handeliodendron bodinieri* and *Eurycorymbus cavaleriei* were submitted to GenBank of National Center for Biotechnology Information (NCBI), where *H. bodinieri* and *E. cavaleriei* were given MK552107 and MK552106 accession numbers, respectively.

### Genome features analysis

Length of the whole chloroplast genome, numbers of genes, and categories of genes were analyzed in Geneious 11.0.4 [58]. MEGA [60] was adopted to calculate the guanine-cytosine (GC) content. A total of 25 species from Sapindaceae were compared in this analysis, including *Aesculus wangii*, *Ae. chinensis*, *Acer. buergerianum* subsp. *ningpoense*, *A. tataricum* subsp. *ginnala*; *A. davidii*, *A. truncatum*, *Acer miaotaiense*, *A. griseum*, *A. wilsonii*, *A. sino-oblongum*, *A. morrisonense*, *A. palmatum*, *Dipteronia dyeriana*, *D. sinensis*, *Dimocarpus longan*, *Dodonaea viscosa*, *Sapindus mukorossi*, *Xanthoceras sorbifolium*, *Litchi chinensis*, *Koelreuteria paniculata*, *Pometia tomentosa*, and *Nephelium lappaceum*. CodonW software (http://codonw.sourceforge.net/culong.html#CodonW) was used for analyzing the codon preference. In the current study, all sequences downloaded from NCBI as well as their corresponding GenBank accession numbers are presented in the Table S1.

### Dispersed repeats and simple sequence repeats detection

Dispersed repeats, including forward, reverse, palindromic, and complement repeat types, were identified online using REPuter program [61]. The minimal repeat size was limited to no less than 30 bp with the Hamming distance equal to three, and with the other settings retained as default. Furthermore, IR regions are the most typical palindromic repeat sequences in the cp genome, hence were not included in the analysis. The simple sequence repeats (SSRs) of *Handeliodendron bodinieri* and *Eurycorymbus cavaleriei* chloroplast genomes were detected using MIcroSAtellite identification tool (MISA) [62]. The parameters were adjusted for the identification of mononucleotide, dinucleotide, tri nucleotide, tetra nucleotide, penta nucleotide and hexa nucleotidemotifs with a minimum of 10, 5, 4, 3, 3, and 3 repeats, respectively.

### Whole cp genome sequence comparisons

The mVISTA program [63] was employed to compare the whole cp genome divergence with related species in Shuffle-LAGAN mode, with the *Eurycorymbus cavaleriei* chloroplast genome as the reference. To explore the highly divergent regions of the cp genomes in the Sapindaceae species, the software DnaSP version 5.1 [64] was used to calculate nucleotide diversity (Pi). The step size and window length were set as 200 bp and 600 bp, respectively. Geneious 11.0.4 [58] was used to detect the contraction/expansion of the inverted repeat regions (IRs), and the final graph of expansions/contractions was visualized using Adobe Illustrator. The genome rearrangement analyses of *H. bodinieri* and *E. cavaleriei* cp genomes were performed using Mauve with default settings [65].

### Selective pressure analysis

To identify the positive selection sites of protein-coding sequences in the cp genome, we calculate the non-synonymous (dN) and synonymous (dS) substitution rates using EasyCodeML v1.12 [66]. The analysis was calculated based on four site models (M0 vs. M3, M1a vs. M2a, M7 vs. M8, and M8a vs.M8) with likelihood ratio test (LRT) threshold of *p* < 0.05 elucidating adaptation signatures within the genome. Bayes Empirical Bayes (BEB) [67] and Naive Empirical Bayes (NEB) analysis were implemented in the M8 model to detect positive selection sites of the selected genes. Each single-copy CDS sequence was aligned under the codon model and then concatenated into one matrix. Subsequently, the ML tree was constructed using IQ-TREE [68] and then used as an input tree. Due to the lack of sufficient Dodonaeoideae cp genome sequences, the family was divided into two clades to carry out the adaptive evaluation analysis based on the classification of Buerki et al. [17], including Dodonaeoideae+Sapindoideae and Hippocastanoideae.

### Phylogenetic analysis

To ascertain the phylogenetic position of *Handeliodendron bodinieri* and *Eurycorymbus cavaleriei* within the Sapindales, a total of 42 species were analyzed, of which 40 complete cp genome sequences were downloaded from NCBI (Table S1). Among these species, *Euscaphis japonica* in the order Crossosomatales was used as outgroup. Phylogenies were constructed by Maximum Likelihood (ML) and Bayesian Inference (BI) analyses using the complete cp genome sequences, coding sequence (CDS), LSC, SSC, and IR regions. For the CDS dataset, we extracted 79 CDSs using Geneious 11.0.4 [58]. Each CDS matrix was aligned individually by MAFFT [69] with the codon model. All alignments were eventually concatenated into one supermatrix by PhyloSuite [70]. ModelFinder [71] was used to select the best-fit model for constructing phylogenetic tree based on the BIC standard. ML analysis was conducted using IQ-TREE with 1000 bootstrap replicates [68], whereas BI analysis were carried out using MrBayes 3.2.2 [72]. For BI analysis, the two independent Markov Chain Monte Carlo (MCMC) analyses were run for 10,000,000 generations. Trees were sampled every 1000 generations, and the first 25% of trees generated were discarded as burn-in. Finally, the phylogenetic trees were viewed and edited using Figtree 1.4 (https://github.com/rambaut/figtree).

## Results

### General characteristics of the cp genomes

**Fig. 1:**
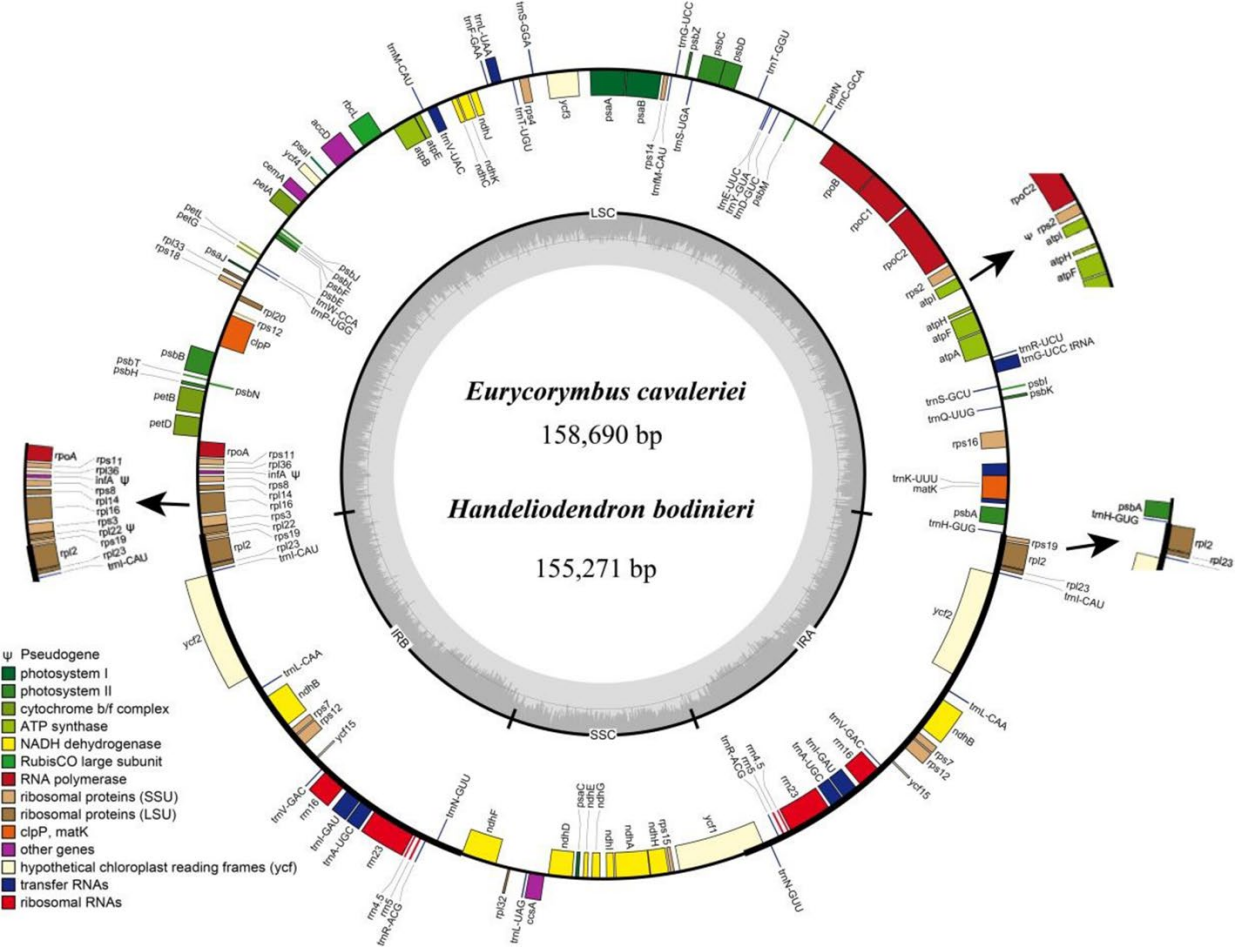
Circular gene map of chloroplast genomes of *Handeliodendron bodinieri* and *Eurycorymbus cavaleriei*. The gray arrowheads indicate the direction of the genes. Genes on the outside and inside of the circle are transcribed in clockwise and counterclockwise directions, respectively. Genes belonging to different functional groups are color coded. The innermost darker gray corresponds to GC content, whereas the lighter gray corresponds to AT content. IR, LSC, SSC indicate inverted repeat, large single copy region, and small single copy region, respectively. The black arrows indicate annotation in *H. bodinieri* that are inconsistent with those in *E. cavaleriei*

Chloroplast genome structures of *Handeliodendron bodinieri* and *Eurycorymbus cavaleriei* are conserved and their cp genome sizes were 151,271 and 158,690 bp, respectively (Fig. 1). Both genomes presented the quadripartite structures including a pair of inverted repeats (IRs) of 25,724 bp and 26,910 bp, a large single copy (LSC) region of 85,092 bp and 86,874 bp, and a small single copy (SSC) region of 15,812 bp and 17,966 bp in *H. bodinieri* and *E. cavaleriei* respectively (Table 1). In the whole genome, the total GC content of *H. bodinieri* and *E. cavaleriei* were 37.8 and 37.9%, respectively. Moreover, the GC content was unevenly distributed in the cp genome of *H. bodinieri* and *E. cavaleriei*. The IR region of *H. bodinieri* showed the highest GC contents (43.1%), followed by 36.0% in the LSC region, whereas the SSC region exhibited the lowest GC content of 31.5%. Similarly, the IR region of *E. cavaleriei* showed higher GC contents (42.8%) than that of the LSC region (36.1%) and SSC region (32.3%). In the coding sequences (CDS), the GC content of *H. bodinieri* and *E. cavaleriei* were 38.1 and 38.4%, respectively.

**Table 1:** Comparison of chloroplast genome feature of *Handeliodendron bodinieri* and *Eurycorymbus cavaleriei*

Species	Location	length (bp)	T (U) (%)	C (%)	A (%)	G (%)	G + C (%)
*H. bodinieri*	LSC	85,092	32.8	18.5	31.1	17.5	36.0
	SSC	18,731	34.2	16.3	34.4	15.1	31.5
	IR	25,724	28.3	20.9	28.6	22.3	43.1
	CDS	78,970	31.4	19.5	30.5	18.6	38.1
	Total	155,271	31.5	19.3	30.6	18.6	37.8
*E. cavaleriei*	LSC	86,874	32.7	18.6	31.3	17.5	36.1
	SSC	17,996	33.8	16.7	33.9	15.6	32.3
	IR	26,910	28.5	20.8	28.6	22.1	42.8
	CDS	79,251	31.3	19.7	30.3	18.7	38.4
	Total	158,690	31.4	19.3	30.7	18.6	37.9

A total of 114 unique genes were annotated in *H. bodinieri* and *E. cavaleriei*, including 77 protein-coding genes (PCGs) in *H. bodinieri*, whereas 79 PCGs were annotated in *E. cavaleriei*. 31 tRNA genes and four rRNA genes were annotated in the two species (Table 2). Among these, three genes (*infA*, *rpl22*, *rpl2*) and one gene (*infA*) had the stop codon appearing prematurely, thus, were annotated as pseudogenes in *H. bodinieri* and *E. cavaleriei*, respectively. In total, 18 genes were duplicated in the IR regions of *H. bodinieri* cp genome, including seven tRNA genes (*trnA-UGC*, *trnI-CAU*, *trnI-GAU*, *trnL-CAA*, *trnN-GUU*, *trnR-ACG*, *trnV-GAC*), four rRNA genes (*rrn4.5*, *rrn5*, *rrn16*, *rrn23*), and seven PCGs (*ndhB*, *rpl2*, *rpl23*, *rps7*, *rps12*, *ycf2*, *ycf15*). For the cp genome of *E. cavaleriei*, seven tRNA genes (*trnA-UGC*, *trnI-CAU*, *trnI-GAU*, *trnL-CAA*, *trnN-GUU*, *trnR-ACG*, *trnV-GAC*), four rRNA genes (*rrn4.5*, *rrn5*, *rrn16*, *rrn23*), and eight PCGs (*ndhB*, *rpl2*, *rpl23*, *rps7*, *rps19*, *rps12*, *ycf2*, *ycf15*) were located in the IR regions.

**Table 2:** Summary of assembled gene functions of *Handeliodendron bodinieri* and *Eurycorymbus cavaleriei* chloroplast genomes

Gene Family	Gene Names
Subunits of ATP synthase	atpA, atpB, atpE, atpF*, atpH, atpI
Subunits of NADH dehydrogenase	ndhA*, ndhB*(×2), ndhC, ndhD, ndhE, ndhF, ndhG, ndhH, ndhI, ndhJ, ndhK
Subunits of cytochrome	petA, petB*, petD*, petG, petL, petN
Subunits of photosystem I	*psaA, psaB, psaC, psaI, psaJ*
Subunits of photosystem II	*psbA, psbB, psbC, psbD, psbE, psbF, psbH, psbI, psbJ, psbK, psbL, psbM, psbN, psbT, psbZ*
Subunit of rubisco	*rbcL*
Subunit of Acetyl-CoA-carboxylase	*accD*
c-type cytochrome synthesis gene	*ccsA*
Envelop membrane protein	*cemA*
Protease	clpP**
Translational initiation	*ψinfA*
Maturase	*matK*
Large subunit of ribosome	rpl2*(×2), rpl14, rpl16*, rpl20, rpl22^*H*^*, rpl23(× 2), rp32, rpl33, rpl36*
DNA dependent RNA polymerase	rpoA, rpoB, rpoC1*, rpoC2
Small subunit of ribosome	*rps2*^*H*^, rps3, rps4, rps7(×2), rps8, rps11, rps12**(× 2), rps14, rps15, rps16*, rps18, rps19(×E)
rRNA Genes	*rrn4.5(×2), rrn5(× 2), rrn16(× 2), rrn23(× 2)*
tRNA Genes	*trnA-UGC(× 2), trnC-GCA, trnD-GUC, trnE-UUC, trnF-GAA, trnfM-CAU, trnG-GCC, trnG-UCC, trnH-GUG, trnI-CAU(× 2), trnI-GAU(× 2), trnK-UUU, trnL-CAA(× 2), trnL-UAA, trnL-UAG, trnM-CAU, trnN-GUU(× 2), trnP-UGG, trnQ-UUG, trnR-ACG(× 2), trnR-UCU, trnS-GCU, trnS-GGA, trnS-UGA, trnT-GGU, trnT-UGU, trnV-GAC(× 2), trnV-UAC, trnW-CCA, trnY-GUA*
Unknown function	ycf1, ycf2(× 2), ycf3**, ycf4, ycf15(× 2)

^*^ Genes containing a single intron; ** Genes containing two introns; (*×* 2) Genes are located within the IR regions and therefore are duplicated; (*×*E) Genes present as two copies in the IR regions of *E. cavaleriei*; ψ indicates a pseudogene; ^H^ Pseudogene in *H. bodinieri* only

Among the annotated genes of *H. bodinieri* and *E. cavaleriei* cp genomes, 18 genes contain introns, including *atpF*, *ndhA*, *ndhB*, *petB*, *petD*, *rpl16*, *rpl2*, *rpoC1*, *rps16*, *trnV-UAC*, *trnL-UAA*, *trnK-UUU*, *trnI-GAU*, *trnG-UCC*, *trnA-UGC*, *clpP*, *rps12*, and *ycf3*. Gene *clpP*, *rps12*, and *ycf3* contain two introns, whereas the other 15 genes have only one intron (Table 3). *Rps12* is a trans-spliced gene with the 5′ end exon located in the LSC region, but the 3′ end in the IR region, as in most other angiosperms. In addition, the longest intron was detected in *trnK-UUU* of both cp genomes, and its length was 2514 bp and 2496 bp, respectively. Similar to other cp genomes, the *matK* gene is located in the intron of *trnK-UUU*.

**Table 3:** Genes with introns in the chloroplast genomes of *Handeliodendron bodinieri* and *Eurycorymbus cavaleriei* as well as the lengths of the exons and introns

Gene	Location	*H. bodinieri*					*E. cavaleriei*				
		Exon I (bp)	Intron I (bp)	Exon II (bp)	Intron II (bp)	Exon III (bp)	Exon I (bp)	Intron I (bp)	Exon II (bp)	Intron II (bp)	Exon III (bp)
*atpF*	LSC	159	756	408			159	750	408		
*clpP*	LSC	69	836	291	645	228	69	853	291	658	228
*ndhA*	SSC	552	1143	540			558	1089	540		
*ndhB*	IR	777	682	756			777	680	756		
*petB*	LSC	6	789	657			6	794	657		
*petD*	LSC	9	740	525			9	677	525		
*rpl16*	LSC	9	1049	402			9	923	402		
*rpl2*	IR	393	661	435			390	664	435		
*rpoC1*	LSC	435	674	1620			435	711	1620		
rps12*	LSC	114	–	232	537	26	114	–	232	533	26
*rps16*	LSC	39	829	225			39	836	225		
*trnV-UAC*	LSC	39	589	37			39	590	37		
*trnL-UAA*	LSC	37	542	49			37	542	49		
*trnK-UUU*	LSC	37	2514	38			37	2496	38		
*trnI-GAU*	IR	42	953	35			42	946	35		
*trnG-UCC*	LSC	23	724	48			23	721	48		
*trnA-UGC*	IR	38	841	35			38	810	35		
*ycf3*	LSC	126	731	228	746	153	126	732	228	767	153

We further compared these basic characteristics of *Handeliodendron bodinieri* and *Eurycorymbus cavaleriei* cp genomes with other genera of Sapindaceae (Table 4). Significantly, we found that the cp genome size of the Hippocastanoideae was generally smaller compared to the other subfamily within Sapindaceae, their size ranged from 152,688 bp (*Acer tataricum* subsp. *ginnala*) to 157,367 bp (*A. palmatum*). Overall, the full-length of cp genome ranged from 152,688 bp (*Acer tataricum* subsp. *ginnala*) to 163,258 bp (*Koelreuteria paniculata*), but the total GC content was similar among 25 cp genomes of Sapindaceae. By comparing single copy regions, we found that *K. paniculata* possessed the largest LSC region with a length of 90,236 bp, whereas *Sapindus mukorossi* possessed the largest SSC region (18,874 bp). Among these cp genomes, the length of IR regions varied from 25,656 bp (*Aesculus chinensis*) to 30,103 bp (*Litchi chinensis*). Interestingly, we also discovered *L. chinensis* presenting the smallest SSC region in these cp genomes. This analysis indicated that the number of rRNA is identical, whereas the number of tRNA (from 37 to 40) and PCGs (83–89) were remarkably similar.

**Table 4:** Statistics on the basic features of the chloroplast genomes from Sapindaceae species

Species	size (bp)	LSC (bp)	IR (bp)	SSC (bp)	GC content (%)	No. rRNA	No. tRNA	No. PCGs
*Acer tataricum* subsp. *ginnala*	152,688	82,529	26,306	17,795	38.2	8	40	87
*Handeliodendron bodinieri*	155,271	85,092	25,724	18,731	37.8	8	37	89
*Aesculus chinensis*	155,528	85,489	25,656	18,727	37.9	8	37	83
*Aesculus wangii*	155,871	84,882	26,390	18,209	38.0	8	40	84
*Acer truncatum*	156,262	86,018	26,086	18,072	37.9	8	40	89
*Acer miaotaiense*	156,595	86,327	26,100	18,068	37.9	8	40	89
*Acer griseum*	156,857	85,227	26,742	18,146	37.9	8	40	86
*Acer buergerianum* subsp. *ningpoense*	156,911	85,314	26,752	18,093	37.9	8	40	89
*Acer davidii*	157,044	85,410	26,761	18,112	37.9	8	40	86
*Acer wilsonii*	157,067	85,418	26,760	18,129	37.9	8	39	88
*Dipteronia dyeriana*	157,071	85,529	26,730	18,082	38.0	8	40	87
*Dipteronia sinensis*	157,080	85,455	26,766	18,093	37.8	8	40	88
*Acer sino-oblongum*	157,118	85,558	26,722	18,119	37.9	8	39	88
*Acer morrisonense*	157,197	85,655	26,728	18,086	37.8	8	40	86
*Acer palmatum*	157,367	85,829	26,689	18,160	37.8	8	40	89
*Eurycorymbus cavaleriei*	158,690	86,874	26,910	17,996	37.9	8	37	87
*Dodonaea viscosa*	159,375	87,204	27,100	17,971	37.9	8	37	88
*Sapindus mukorossi*	160,481	85,649	27,979	18,874	37.7	8	39	88
*Pometia tomentosa*	160,818	85,666	28,396	18,360	37.9	8	37	88
*Dimocarpus longan*	160,833	85,708	28,428	18,269	37.8	8	37	87
*Xanthoceras sorbifolium*	161,231	85,299	28,620	18,692	37.7	8	38	86
*Nephelium lappaceum*	161,356	86,009	28,597	18,153	37.8	8	37	87
*Litchi chinensis*	162,524	85,750	30,103	16,568	37.8	8	37	87
*Koelreuteria paniculata*	163,258	90,236	27,377	18,268	37.3	8	37	85

#### Chloroplast repeated sequences and SSRs

**Fig. 2:**
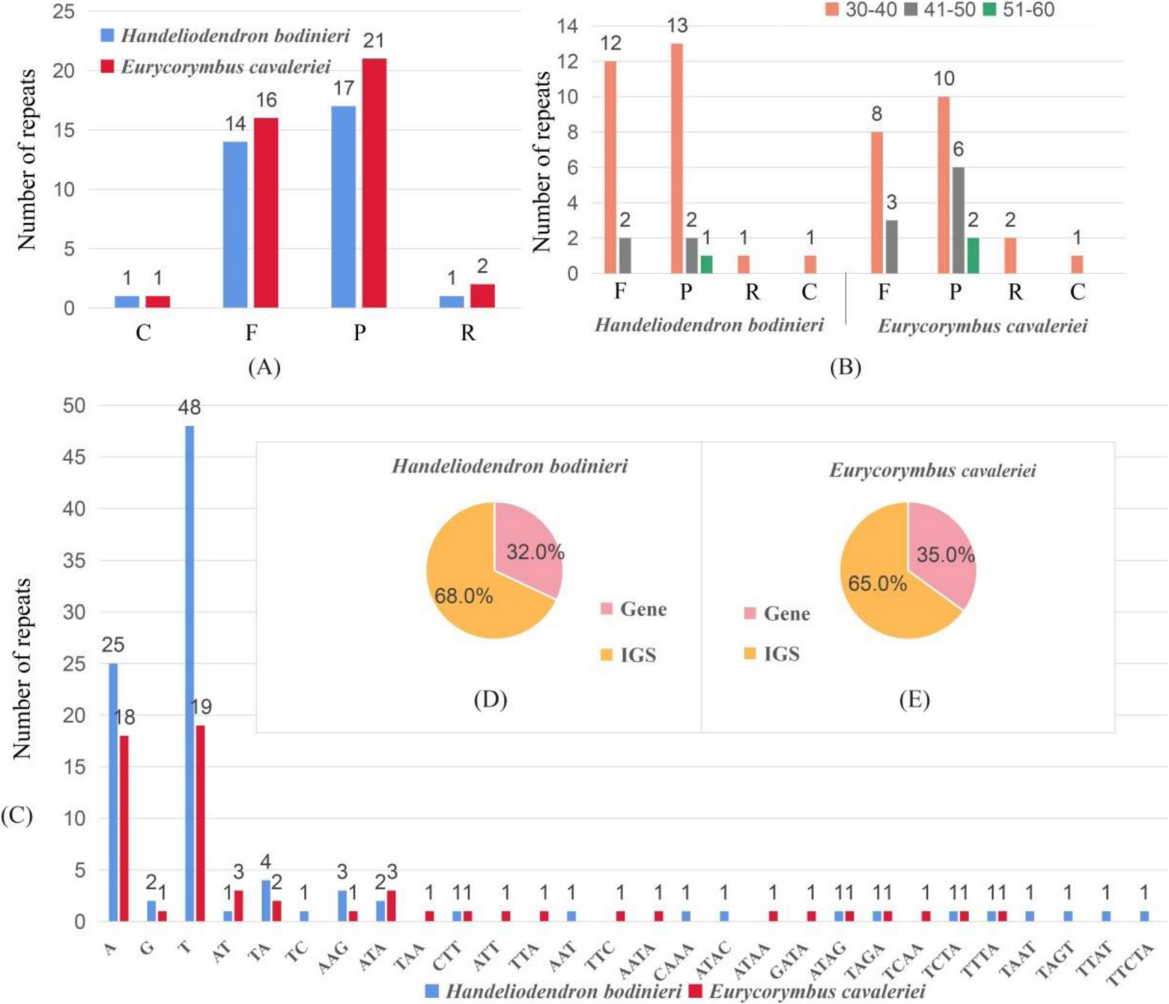
Analyses of repeated sequences in *Handeliodendron bodinieri* and *Eurycorymbus cavaleriei* complete chloroplast genomes. **A** Total of dispersed repeats, the F, R, P, C indicate forward, reverse, palindromic, and complement repeats, respectively; **B** Frequency of dispersed repeats by length; **C** Numbers and types of SSR in the chloroplast genomes of *H. bodinieri* and *E. cavaleriei*; **D** SSR distribution between Gene and intergenic spacer regions (IGS) of *H. bodinieri*; **E** SSR distribution between Gene and intergenic spacer regions (IGS) of *E. cavaleriei*

In the current study, a total of 32 and 39 repeat sequences were detected in *Handeliodendron bodinieri* and *Eurycorymbus cavaleriei* cp genomes, respectively. In cp genome of *H. bodinieri*, there were 14 forward (F), 1 reverse (R), 16 palindromic (P), and 1 complement (C) repeats (Fig. 2, A). For *E. cavaleriei* cp genome, the number of the F, R, P, and C repeats was 16, 2, 20, and 1, respectively. We found that the length of repeat sequences ranged from 30 to 51 bp in *H. bodinieri*, 30–72 bp in *E. cavaleriei* (Fig. 2, B). In total, the results revealed that the P and F repeats were most abundant in all these repeat sequences, and most of palindromic and forward repeats were with 30–40 bp in length.

Here, we observed the simple sequence repeats of the *H. bodinieri* and *E. cavaleriei* cp genomes. The total number of the SSRs was 98 in *H. bodinieri*, whereas 60 in *E. cavaleriei* (Table S2; Fig. 2, C). In *H. bodinieri*, we detected five categories of SSRs, including mononucleotide, dinucleotide, tri nucleotide, tetra nucleotide, and penta nucleotide repeats. Additionally, none of hexa-nucleotides were detected in *H. bodinieri* cp genome. The number of mononucleotide, dinucleotide, tri nucleotide, tetra nucleotide, and penta nucleotide repeats were 75, 6, 7, 9, and 1, respectively (Fig. 2, C). The finding not only showed that the mononucleotide repeats were the most abundant in the cp genome, but also had an outstanding base preference, mainly consist of A or T. Notably, five SSRs were identified in the *ycf1* gene of *H. bodinieri* cp genome, consisting of mononucleotide repeats that contain four poly (T) and one poly (A). In total, there were four types of SSRs in the *E. cavaleriei* cp genome, including mononucleotide, dinucleotide, tri nucleotide, and tetra nucleotide repeats (Table S3). The number of mononucleotide, dinucleotide, tri nucleotide, tetra nucleotide, and penta nucleotide repeats were 38, 5, 8, and 8, respectively. Among these SSRs, the most dominant of SSRs were A or T mononucleotides. We also observed four SSRs in the gene *ycf1*, consist of mononucleotide repeats. Within *H. bodinieri* and *E. cavaleriei* cp genomes, most SSRs were located in the intergenic spacer regions (IGS) (Fig. 2, D and E).

#### Relative synonymous codon usage analysis

**Fig. 3:**
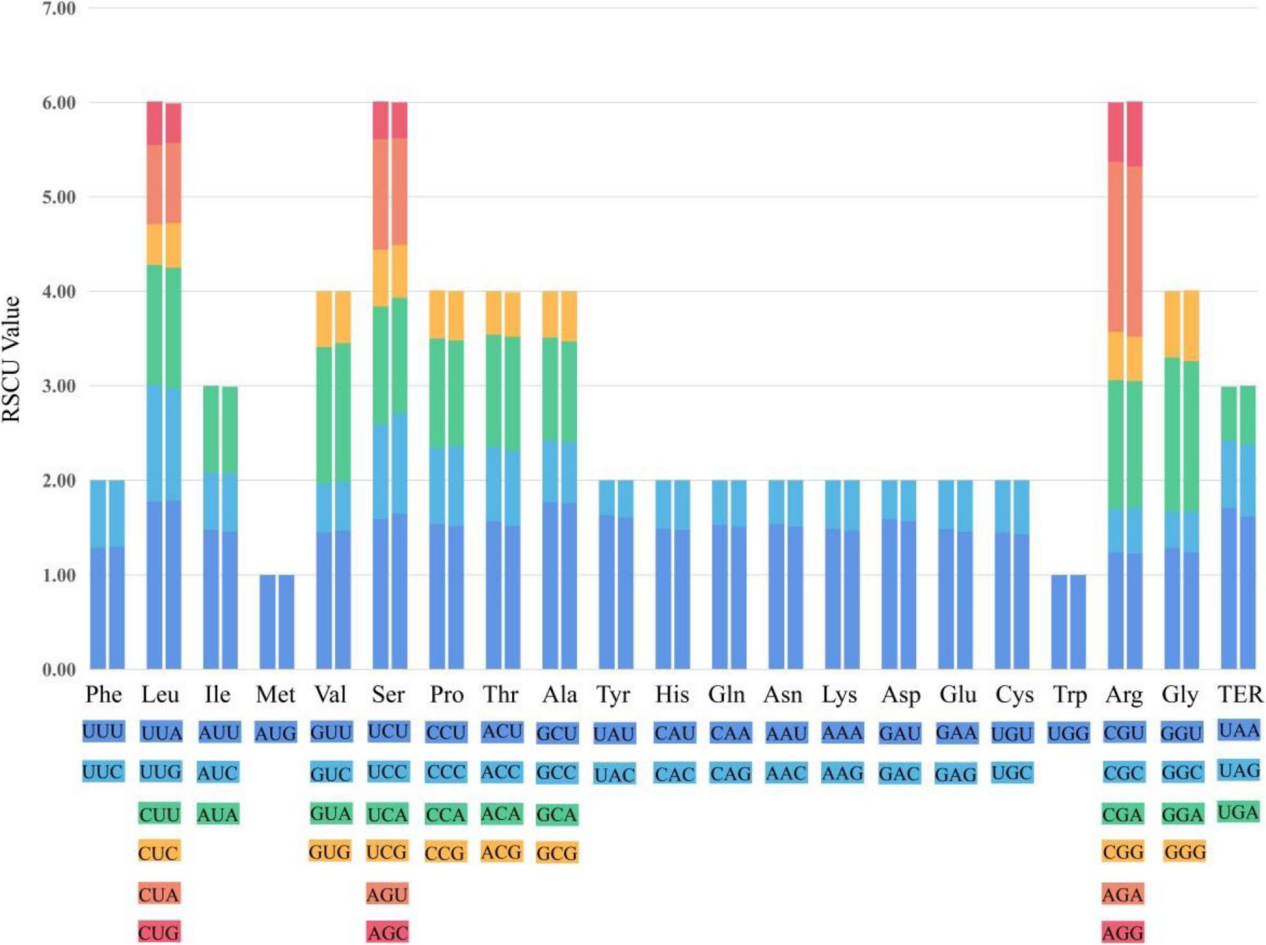
Codon contents of 20 amino acids and stop codons in all coding sequences of the *Handeliodendron bodinieri* and *Eurycorymbus cavaleriei* chloroplast genomes. The histogram on the left side presents each amino acid codon usage within *H. bodinieri* cp genome, and the right side denotes that of within *E. cavaleriei* cp genome. The colour of the histogram corresponds to the colour of codons

The total number of the codons was 26,028 in *Handeliodendron bodinieri*, 26,445 in *Eurycorymbus cavaleriei*. Among the codons, the number of the amino acids less than 1000 was tyrosine (Tyr), glutamine (Gln), histidine (His), methionine (Met), tryptophan (Trp), cysteine (Cys) in both the *H. bodinieri* (Table S4) *and E. cavaleriei* (Table S5). The leucine (Leu) was the most amino acid encoded in the analysis, accounting for 10.5 and 10.6% on average of all amino acids in the *H. bodinieri and E. cavaleriei* cp genomes, respectively. However, the Cys has the lowest number of codons in both the *H. bodinieri and E. cavaleriei* cp genomes excluding the stop codons. The codon usage frequency and relative synonymous codon usage (RSCU) were summarized in Fig. 3. In *H. bodinieri* cp genome, 30 codons had RSCU values more than 1.00, and they all ended with A or U excluding UUG. For the *E. cavaleriei*, there were 31 codons with RSCU values more than 1.00, 29 of which ended with A or U codons, whereas two ended with C and G codons (UCC and UUG). Moreover, we discovered that the RSCU values of three codons (AUG, UGG, and UCC) are 1.00 in the *H. bodinieri* cp genome, while only two codons (AUG and UGG) in *E. cavaleriei* cp genome.

### Comparative chloroplast genomic analysis

**Fig. 4:**
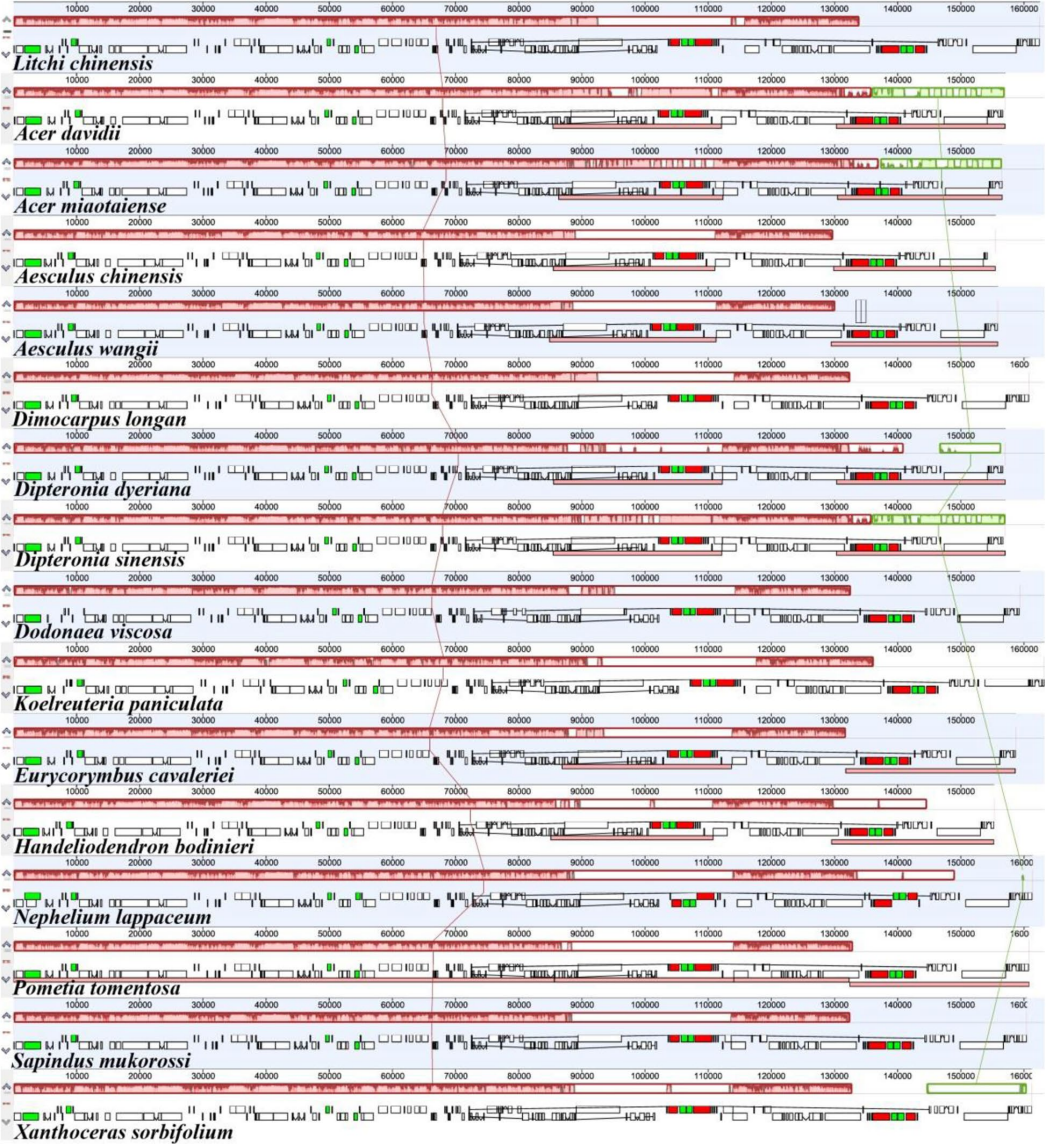
Genomic rearrangement of the 16 Sapindaceae chloroplast genomes, with *Litchi chinensis* set as a reference genome

To identify the sequence divergence of *Handeliodendron bodinieri* and *Eurycorymbus cavaleriei* cp genomes, the genomic rearrangement was detected, with *Litchi chinensis* as the reference (Fig. 4). In total, 16 cp genomes of 13 genera from Sapindaceae were used for analysis. Comparative analysis showed that all cp genomes were highly conserved, an indication that inversion and translocation in genes or plastid segments was not detected in the final results.

**Fig. 5:**
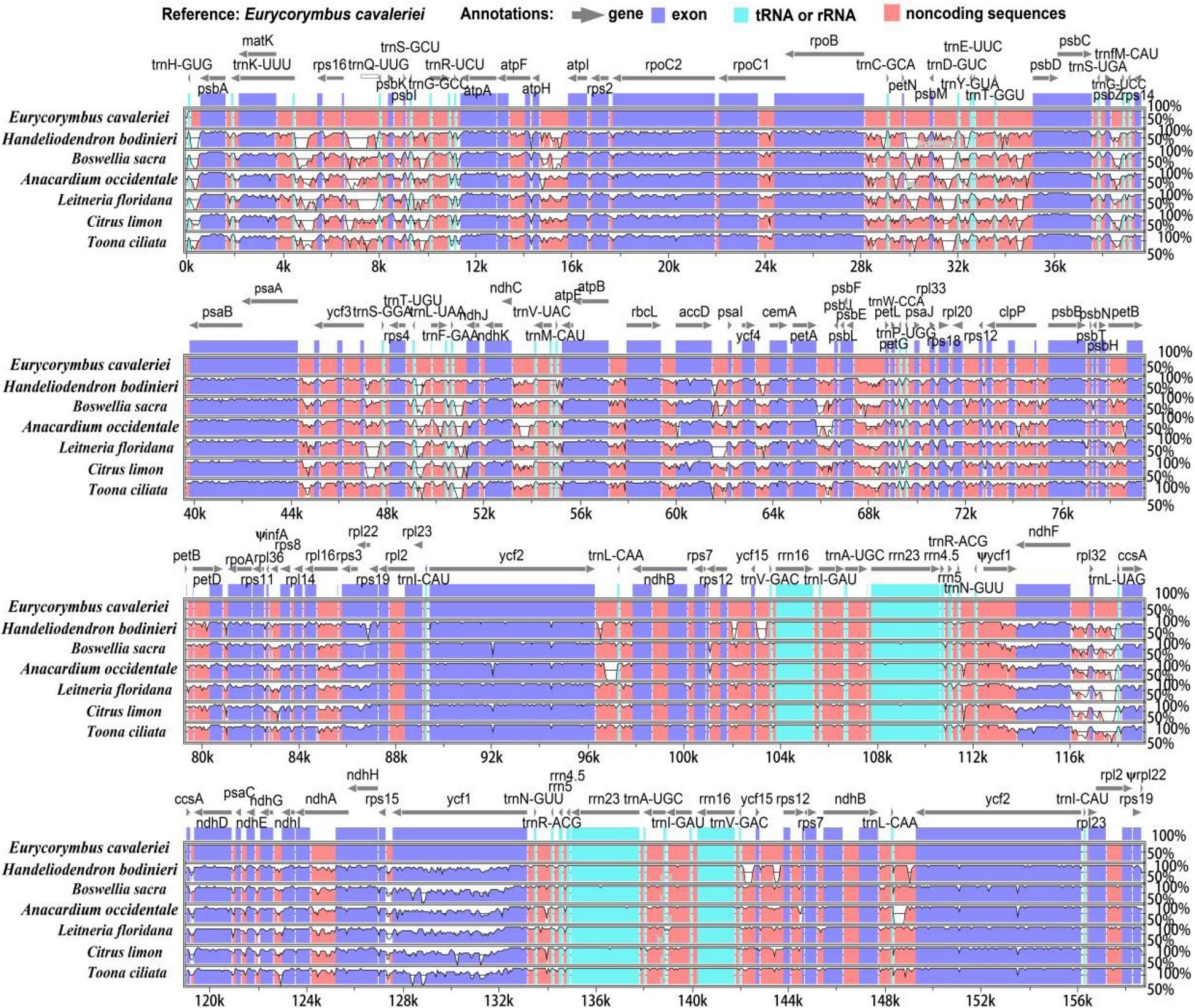
Visualization of genome alignment of the complete genome of seven complete chloroplast genomes from Sapindales. The cp genome of *Eurycorymbus cavaleriei* is used as the reference. X-axis indicates the sequence coordinates in the whole cp genome. Y-axis represents the similarity of the aligned regions, indicating percent identity to the reference genome (50–100%)

In addition, we performed multiple sequence alignment of the complete chloroplast genome sequences from different families in Sapindales with *E. cavaleriei* as the reference (Fig. 5). The comparison analyses revealed that coding regions were more conserved than the non-coding regions, and the SSC and LSC regions exhibited more variation than IR regions in all cp genomes. Moreover, there were almost no variation in four rRNA genes within LSC regions, and the genes were highly conserved. A total of five genes, including *matK*, *accD*, *ycf1*, *ndhF*, and *rpl22*, were detected the most divergent in these cp genomes. As shown in Fig. 5, the significant variations were detected in the intergenic regions of the LSC and SSC, including *trnH-psbA*, *trnk-rps16*, *rps16-trnQ*, *psbM-trnY*, *psbZ-trnG*, *trnL-trnF*, *trnF-ndhJ*, and *rpl32-trnL*.

#### Divergence hotspot identification

**Fig. 6:**
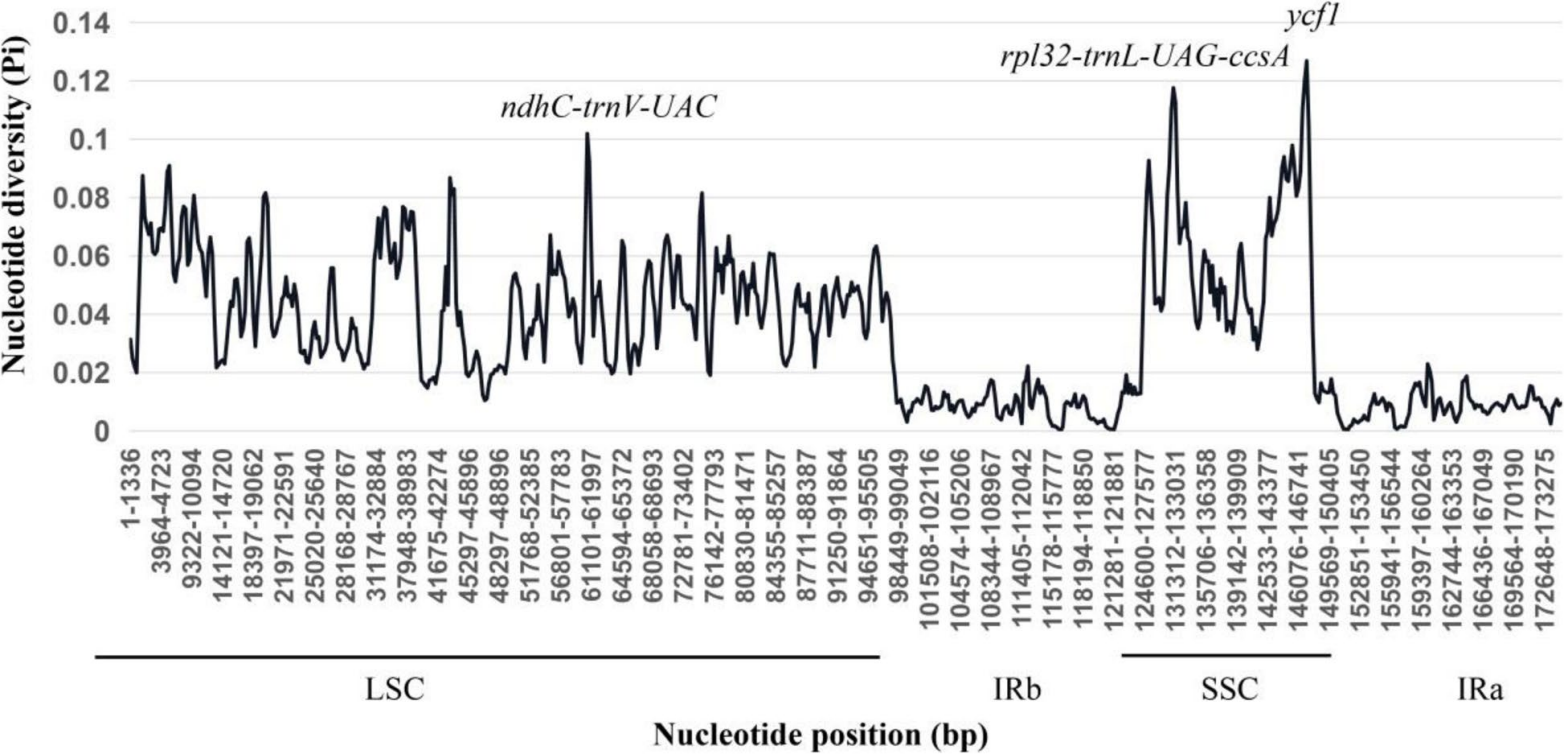
Sliding window analysis based on the 13 cp genome sequences of Sapindaceae. X-axis: position of the Midpoint of a window; Y-axis: Nucleotide diversity of each window (Pi)

To further estimate the divergence of cp genomes among the Sapindaceae species, a total of 11 cp genome sequences of other genera were chosen randomly to calculate nucleotide diversity (Pi), including *Acer davidii*, *A. miaotaiense*, *Aesculus chinensis*, *Ae. wangii*, *Dimocarpus longan*, *Dipteronia dyeriana*, *D. sinensis*, *Dodonaea viscosa*, *Litchi chinensis*, *Nephelium lappaceum*, *Pometia tomentosa*, *Sapindus mukorossi*, and *Xanthoceras sorbifolium*. Based on this analysis, we identified three remarkably divergent regions among these complete cp genomes, which included *ndhC-trnV-UAC*, *rpl32-trnL-UAG-ccsA*, and *ycf1* (Fig. 6). Gene *ycf1* is the most divergent region with the highest Pi value (0.127) and is located in the SSC region, whereas *ndhC-trnV-UAC* and *rpl32-ccsA* intergenic spacers were located in LSC regions. These highly divergent regions could be used as potential molecular markers for phylogenetic reconstruction of the family Sapindaceae. Overall, the result of this study revealed that sequence divergence was concentrated in the LSC and SSC regions, whereas IR regions presented less divergence, consistent with the mVISTA results (Fig. 5).

#### Expansion and contraction of IRs

**Fig. 7:**
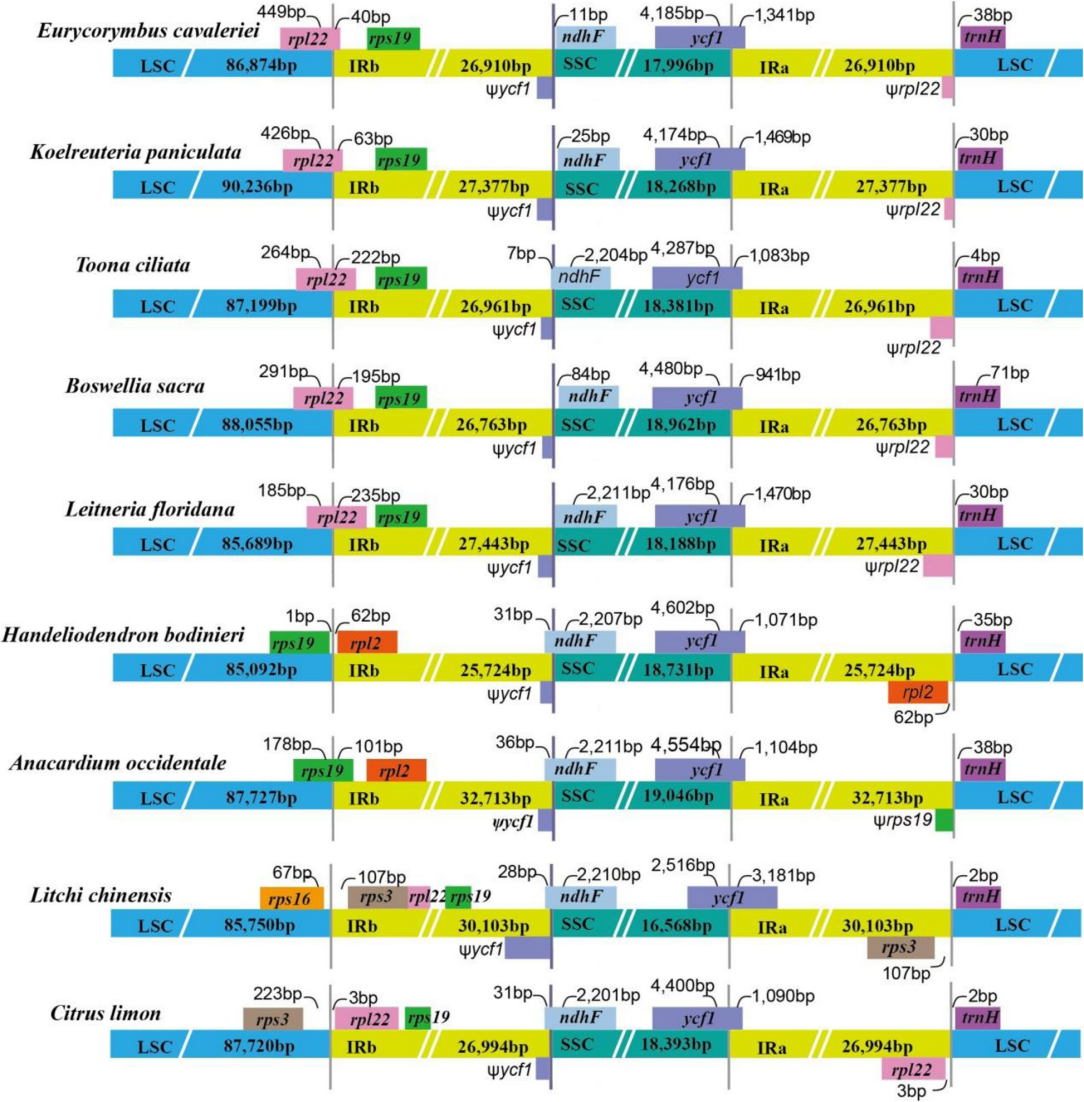
Comparison of the Large Single-Copy (LSC), Small Single-Copy (SSC), and inverted repeat (IR) boundary regions across nine Sapindales chloroplast genomes, ψ indicates a pseudogene. The mumbers next to gene indicate the distance to the boundary, or the length of these genes in single copy regions and inverted repeat regions

We compared the single-copy (SC) and inverted repeat (IR) boundary region among different families within the order Sapindales to find potential evolutionary events. A total of nine cp genomes were chosen randomly, *Koelreuteria paniculata* (Sapindaceae), *Litchi chinensis* (Sapindaceae), *Leitneria floridana* (Simaroubaceae), *Toona ciliata* (Meliaceae), *Anacardium occidentale* (Anacardiaceae), *Boswellia sacra* (Burseraceae), *Citrus limon* (Rutaceae), as well as our newly sequenced species (Fig. 7). The SSC/IRa boundary located in the coding region of gene *ycf1* in all cp genomes, with 2516 bp to 4602 bp in SSC region. The gene *ycf1* has the largest fragment in SSC region of *H. bodinieri*. In five cp genomes, including *H. bodinieri*, *L. chinensis*, *T. ciliata*, *A. occidentale*, and *C. limon*, the gene *ndhF* spanned IRb/SSC boundary, with 7–36 bp in the IRb region. However, the gene *ndhF* was wholly located in the IRb region of four cp genomes (*E. cavaleriei*, *K. paniculata*, *L. floridana*, and *B. sacra*), which was separated from the IRb/SSC border by a spacer varying from 0 to 84 bp. The gene *ycf1* in the border region between IRb and SSC is treated as a pseudogene because of the incomplete duplication of the normal copy. Similarly, *rpl22* and *rps19* genes in IRa region near the IRa/LSC boundary region, and was annotated as a pseudogene, including *E. cavaleriei*, *K. paniculata*, *T. ciliata*, *B. sacra*, *L. floridana*, and *A. occidentale*. In all cp genomes, there was significant variation in the LSC/IRb boundary regions. The LSC/IRb boundary was crossed by the gene *rpl22* in five cp genomes, and the length of the *rpl22* fragment located in the LSC region ranged from 185 bp (*L. floridana*) to 449 bp (*E. cavaleriei*). The *rpl22* was entirely located in the IRb region of *C. limon* cp genome. In *H. bodinieri* cp genome, *rps19* and *rpl2* genes were entirely located within the LSC and IRb region near the LSC/IRb boundary, respectively. In *A. occidentale* cp genome, we also found that *rps19* gene was located in the LSC/IRb boundary, and 178 bp extended into the LSC region. In *L. chinensis*, the *rps16* and *rps3* genes in the near LSC/IRb were located in LSC and IRb regions, respectively. In the IRa/LSC boundary regions, gene *trnH* was completely located in LSC region of all cp genomes, which was 0–38 bp away from the IRb/SSC boundary. In a word, IR regions of the *H. bodinieri* showed a significant contraction, whereas it presented a notable expansion in *E. cavaleriei* cp genome.

#### Selective pressure analysis

In the present study, we randomly chose different genera from Dodonaeoideae, Sapindoideae, and Hippocastanoideae. The Dodonaeoideae + Sapindoideae clade contains eight species, including *Nephelium lappaceum*, *Sapindus mukorossi*, *Litchi chinensis*, *Dodonaea viscosa*, *Koelreuteria paniculata*, *Dimocarpus longan*, *Pometia tomentosa*, and *Eurycorymbus cavaleriei*, while the Hippocastanoideae clade contains five species, including *Dipteronia sinensis*, *Aesculus wangii*, *Ae. chinensis*, *Acer morrisonense*, and *Handeliodendron bodinieri*. The analysis of Dodonaeoideae + Sapindoideae clade showed that six genes in the five cp genomes are under significant positive selection (Table S6), including *clpP*, *ndhF*, *petA*, *rpoC1*, *rpoC2*, and *rps11*. In the NEB method, a total of five genes exhibited under positive selection in the cp genome of Hippocastanoideae, while the BEB method showed six genes were under significant positive selection (Table S7). In these analyses of Hippocastanoideae and Dodonaeoideae + Sapindoideae, we identified that three sites were detected as sites of positive selection, which were distributed in gene *ndhF* that related to photosynthesis. In the analysis of Hippocastanoideae, the gene *rpoC2* harbored 23 and 32 sites under positive selection according to the NEB and BEB methods, respectively. In addition, we found that the gene *ycf1* has ten positive selection sites based upon two methods.

### Phylogenetic analysis of Handeliodendron bodinieri and Eurycorymbus cavaleriei within Sapindales

**Fig. 8:**
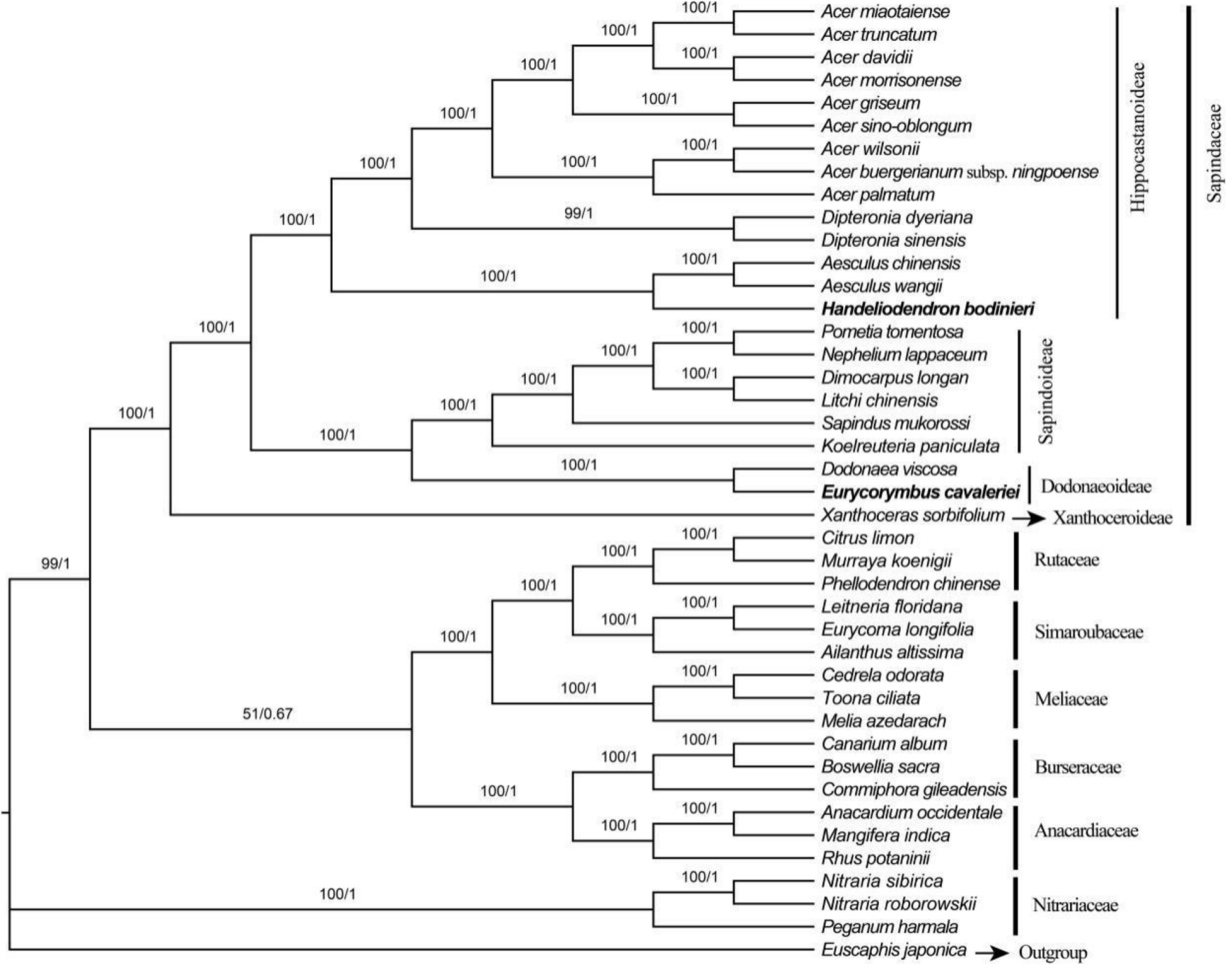
Phylogenetic tree reconstruction of Sapindales using the maximum likelihood (ML) and Bayesian inference (BI) method based on complete chloroplast genome sequences. Only the ML tree is shown, because its topology is identical to that of the obtained BI tree. ML supports/ BI posterior probabilities values are indicated on the nodes

**Fig. 9:**
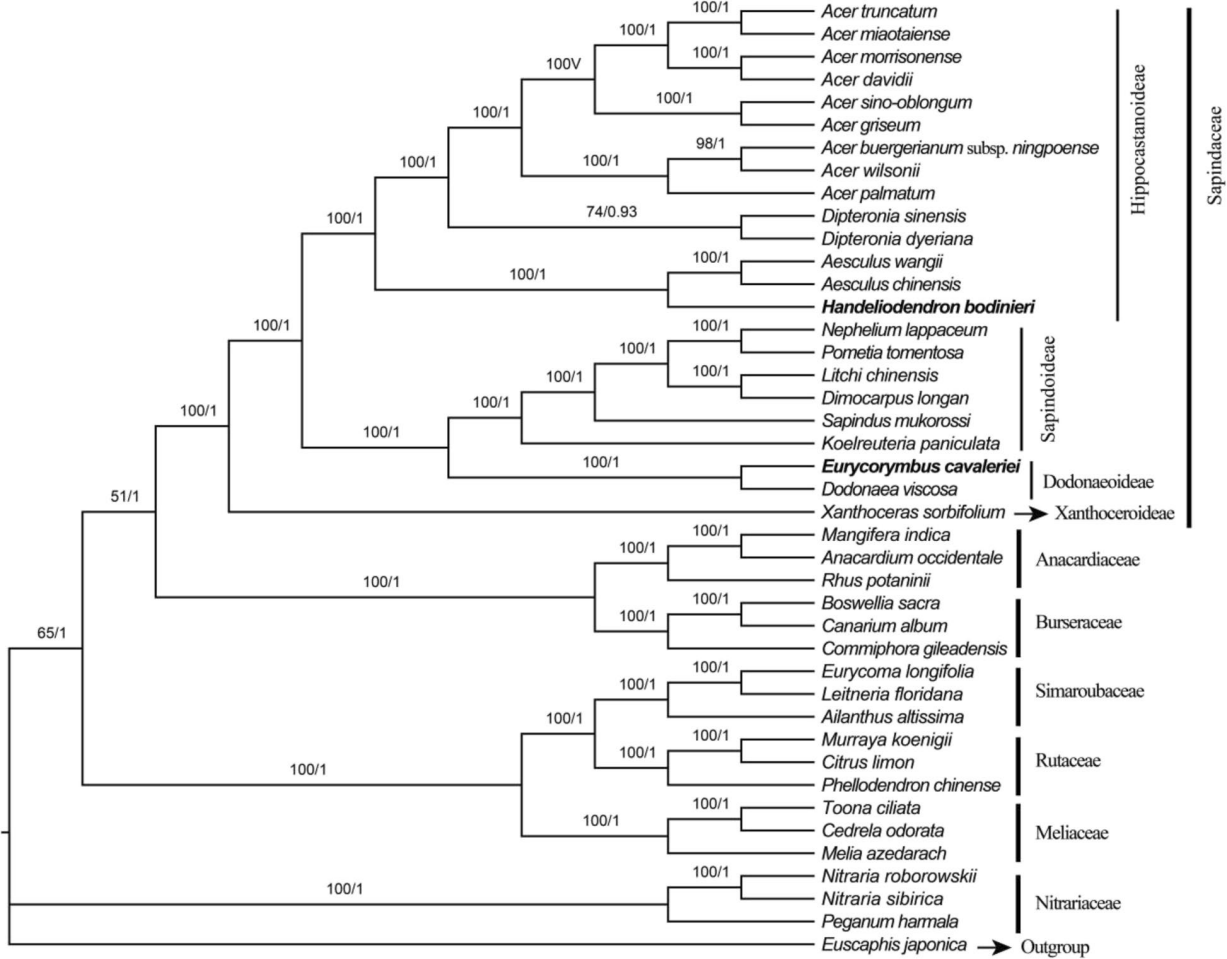
Phylogenetic tree reconstruction of Sapindales using the maximum likelihood (ML) Bayesian inference (BI) method based on 79 coding sequences. Only the ML tree is shown, because its topology is identical to that of the obtained BI tree. ML supports/BI posterior probabilities values are indicated on the node

In this study, phylogenetic analyses were performed based on different datasets, including complete chloroplast genome sequences, coding sequence (CDS), LSC, SSC, and IR regions, with corresponding results in Fig. 8, Fig. 9, Fig. S1, Fig. S2, and Fig. S3, respectively. Detailed information of best-fit model for ML and BI tree are listed in Table S8. All phylogenetic trees consistently revealed that *Handeliodendron bodinieri* is sister to the clade consisting of *Aesculus chinensis* and *Aesculus wangii* (BS = 100, PP = 1.00), strongly support *Eurycorymbus cavaleriei* being sister with *Dodonaea viscosa* (BS = 100, PP = 1.00).

## Discussion

### Features of complete chloroplast genome and comparative Analyses

GetOrganelle is a state-of-the-art toolkit to accurately assemble organelle genomes from whole-genome sequencing data [50]. In the current study, GetOrganelle was utilized to assemble the complete chloroplast genome sequences of *Handeliodendron bodinieri* and *Eurycorymbus cavaleriei* based on the newly sequenced Illumina data. The total size of *H. bodinieri* cp genome is 158,690 bp, which is different from the previous reports of Chen et al. [47] and Du et al. [48]. The full-length of *H. bodinieri* cp genome is 151,271 bp and is also different from that of the previous report [49]. Their genome exhibited a typical quadripartite structure, including a pair of inverted repeats (IRs), a large single copy (LSC) region, and a small single copy (SSC) region, which was the same as that reported for most other angiosperms [73–75]. This study revealed that gene content and gene order in *H. bodinieri* and *E. cavaleriei* cp genomes were quite similar to that of other published Sapindaceae species [76–78]. Additionally, GC content was unevenly exhibited in the cp genome of *H. bodinieri* and *E. cavaleriei* and the IR region showed higher GC contents than that of the LSC region and SSC region, which may be attributed to four rRNA genes with low A/T content. Within the *H. bodinieri* and *E. cavaleriei* cp genomes, the *clpP*, *rps12*, and *ycf3* genes possess one intron, and 15 genes contain two introns. Pseudogenization of the gene *infA* was has been detected in *H. bodinieri* and *E. cavaleriei* cp genomes, the same results were observed in those of other Sapindaceae species [79–81]. Besides, we detected the pseudogenization of *rpl22* and *rpl2* genes in *H. bodinieri* cp genome and the latter was also annotated as pseudogene in that of *Acer takesimense* [82]. A detailed comparative analysis of the complete chloroplast genomes revealed that genomic structure, gene content, PCGs, and total GC content were remarkably similar or identical within 11 genera from Sapindaceae, which were consistent with most studies [83–85]. Significantly, Hippocastanoideae generally have a smaller cp genome size compared to other subfamilies. Considering that not all published cp genome sequences of Sapindaceae species were listed in this table, we further observe all Sapindaceae cp genome sequences in GeneBank database, and the final results supported this finding. However, the work of Luo et al. [86] revealed that the cp genome of *Acer coriaceifolium* is 159,736 bp in size (Accession: SY9280, it was not be released), and the base composition was asymmetric with an overall GC content of 43.00%. The length of *A. coriaceifolium* cp genome was bigger than that of published Dodonaeoideae species, which was not consistent with the above finding. Meanwhile, we also found that there are three *A. coriaceifolium* cp genome sequences with 155,944 bp in the GeneBank database (Accession: MW067038, NC_050669, and MN315271). Overall, the Hippocastanoideae species have a smaller cp genome size and exhibit a close relationship to some extent, although this finding should be tested further with sufficient sampling.

The study demonstrated that palindromic (P) and forward (F) repeats were the most abundant in all dispersed repeats, and most of them were 30–40 bp in length, similar to previous studies [33, 87–89]. Simple sequence repeats (SSRs) were widely used as a molecular marker for studies of genetic diversity and population structure [90–92]. We observed five and four types of SSRs in *H. bodinieri* and *E. cavaleriei* cp genomes, respectively. Among these SSRs, the most dominant of SSRs were A or T mononucleotides. Furthermore, most of SSRs were in the intergenic spacer regions (IGS), which is consistent with other studies [80, 93]. The finding not only showed that the mononucleotide repeats were the most abundant in the cp genome but also had an A/T base preference.

Within *Handeliodendron bodinieri* and *Eurycorymbus cavaleriei* coding sequences, the leucine (Leu) was the frequent amino acid, while the least abundant amino was cysteine (Cys) excluding the stop codons. Moreover, the findings of this study revealed that most codons ended with A or U when RSCU values more than 1.00. As a common phenomenon in cp genomes of plants, similar results have been reported in previous studies [74, 94–96].

The mVISTA results revealed that coding regions were more conserved than the non-coding regions, and the SSC and LSC regions exhibited more variation than IR regions in all cp genomes. The results of this study are consistent with previous finings in other species [29, 75, 76, 97]. In total, we identified five genes present significant variations in these cp genomes, such as *matK*, *accD*, *ycf1*, *ndhF*, and *rpl22*. Additionally, eight intergenic regions, namely *trnH-psbA*, *trnk-rps16*, *rps16-trnQ*, *psbM-trnY*, *psbZ-trnG*, *trnL- trnF*, *trnF-ndhJ*, and *rpl32-trnL* also present significant variations. The nucleotide diversity tests indicate IR regions showed relatively low diversity, but we found *ndhC-trnV-UAC*, *rpl32-trnL-UAG-ccsA*, and *ycf1* were remarkably divergent regions in LSC and SSC regions, which could be used as the specific DNA barcodes for Sapindaceae species. Gene *ycf1* was a high variable genic region in cp genome of plants [23, 73, 94], which has abundant SSRs in our newly sequenced species.

Up to date, cp genome sequences of 13 genera from Sapindaceae are deposited in GenBank database (Table 4). Comparative analysis showed that *Handeliodendron bodinieri* and *Eurycorymbus cavaleriei* cp genomes exhibited highly conserved, and the phenomenon of inversion and translocation in genes or plastid segments was not detected in both species. The border regions of cp genome quadripartite structure exhibited expansion and contraction variation, which were common phenomenons in the evolutionary history of land plants [96]. We found that there some significant variation in LSC and IR boundary regions. In the family Sapindaceae, the LSC/IRb boundary was traversed by the gene *rpl22* in four cp genomes, but it is different from those of *H. bodinieri* and *Litchi chinensis*. Interestingly, their boundaries were similar to that of the member of Simaroubaceae (*Leitneria floridana*). We noticed that IRa/LSC boundary regions showed some differences, but gene *trnH* was completely located in LSC region of all cp genomes. In different families, the SSC/IRa and IRb/SSC boundary regions exhibit highly conserved, which resembles most previous studies [98–100]. The pseudogenes, *ycf1* and *rpl22* were present at the IRb/SSC and IRa/LSC boundaries of *E. cavaleriei* cp genome, respectively, because the incomplete duplication of the normal copy. The finding revealed the IR boundary regions of *E. cavaleriei* cp genome are similar to the *K. paniculata*, *T. ciliata*, *B. sacra*, and *L. floridana*, while the IR boundary regions of *H. bodinieri* cp genome resemble that of other reported *Acer* species (*Acer miaotaiense*, *A. sterculiaceum*, *A. amplum*) [46, 80]. IR regions of the *H. bodinieri* and *E. cavaleriei* cp genomes showed noticeable contraction and expansion, respectively. The non-synonymous (dN) and synonymous (dS) substitution rates are useful for inferring the adaptive evolution of genes [24, 101, 102]. We compared different genera from three subfamilies, and the analysis of selection pressure showed that there are a few positively selected genes, which are essential in unfolding evolutionary history of these genera.

### Phylogenetic analysis

Phylogenetic analysis based on different datasets consistently demonstrated that *Handeliodendron bodinieri* was sister to the clade that consisted of *Aesculus chinensis* and *A. wangii* (PP = 1.00, BS = 100). On the basis of plastid *matK* and *rbcL* DNA sequences, the work of Harrington et al. [15] strongly supported *H. bodinieri* was sister to *Aesculus* plus *Billia*. Subsequently, based on a combination of nuclear (ITS) and plastid (*matK*, *rpoB*, *trnD-trnT*, *trnK-matK*, *trnL-trnF*, and *trnS-trnG*) markers, Buerki et al. [18] suggested the possible paraphyly of the *Aesculus* because *Handeliodendron* and *Billia* were nested within it. Our results strongly supported that *H. bodinieri* had a close relationship with the *Aesculus*, which was consistent with the works of Harrington et al. [15] and Buerki et al. [18]. However, the present analyses could not demonstrate the paraphyly of the *Aesculus* because of lacking sufficient samples. In this study, we found *Eurycorymbus cavaleriei* as sister to *Dodonaea viscosa* with strong support (PP = 1.00, BS = 100), which similar to previous studies [45, 47, 48, 76, 84]. Buerki et al. [17, 18] conducted molecular phylogenetic studies of Sapindaceae s. lat. Based on comprehensive sampling. Their results demonstrated that *Eurycorymbus* belongs to a member of the *Dodoneae* group within Dodonaeoideae, strongly supported *Euphorianthus longifolius* as sister to *E. cavaleriei*, but the *E. longifolius* was not sampled here. Furthermore, for the Sapindaceae, it remains ambiguous in terms of the phylogenetic relationship to other members within Sapindales. In whole cp genome analysis, Sapindaceae formed a sister of the Rutaceae + Simaroubaceae + Meliaceae + Burseraceae + Anacardiaceae clade (BS = 99, PP = 1, Fig. 9), but the clade consist of Rutaceae + Simaroubaceae + Meliaceae + Burseraceae + Anacardiaceae was weakly supported (BS = 51, PP = 0.67). In the CDS analysis, Sapindaceae was sister to Anacardiaceae and Burseraceae with weak support (BS = 51, PP = 1, Fig. 9), the result is consistent with previous studies [99, 103]. In the LSC analysis, Sapindaceae was sister to the Burseraceae + Anacardiaceae + Nitrariaceae clade (BS = 49, PP = 0.99, Fig. S1), In the SSC analysis, Sapindaceae was sister to the Rutaceae + Simaroubaceae + Meliaceae clade (BS = 52, PP = 1, Fig. S2), which is similar to the previous work [84, 85]. Overall, as the sister group of Sapindaceae, most results exhibited weak support in maximum likelihood analysis. This work will contribute to a comprehensive understanding of plastome evolution in Sapindaceae species and provide valuable chloroplast genomic information for further elucidating the circumscription of Sapindaceae at the cp genome level.

## Conclusion

In this work, we sequenced and assembled the complete chloroplast genome of *Handeliodendron bodinieri* and *Eurycorymbus cavaleriei*. Their gene order, gene content, and molecular structure are similar to that of cp genomes of other Sapindaceae species. Comparative analysis of complete cp genomes revealed that the cp genome size of the Hippocastanoideae was generally smaller across Sapindaceae. We detected three highly divergent regions, which could be used as the specific DNA barcodes within Sapindaceae. Phylogenetic results consistently confirm that *H. bodinieri* has a close relationship with the genus *Aesculus*, strongly support *E. cavaleriei* as sister to *Dodonaea viscosa*. As the national-level protected species, both *H. bodinieri* and *E. cavaleriei* attract scientific attention in many aspects, thus this work will provide valuable chloroplast genomic information, and contribute to facilitating future studies in population genetics and conservation biology.

## Supplementary Information

**Additional file 1: Table S1.** All sequences that were used for understanding the phylogenetic analysis within Sapindales, including their corresponding CDS information and GenBank accession numbers.

**Additional file 2: Table S2.** Simple sequence repeats (SSRs) in the *Handeliodendron bodinieri* chloroplast genome.

**Additional file 3: Table S3.** Simple sequence repeats (SSRs) in *Eurycorymbus cavaleriei* chloroplast genome.

**Additional file 4: Table S4.** Relative synonymous codon usage (RSCU) of *Handeliodendron bodinieri* chloroplast genome.

**Additional file 5: Table S5.** Relative synonymous codon usage (RSCU) of *Eurycorymbus cavaleriei* chloroplast genome.

**Additional file 6: Table S6.** Positive selected sites detected in the cp genome of the Dodonaeoideae + Sapindoideae. **Table S7.** Positive selected sites detected in the cp genome of the subfamily Hippocastanoideae.

**Additional file 7: Table S8.** Best-fit Models in ML and BI analysis.

**Additional file 8: Figure S1.** Phylogenetic tree reconstruction of Sapindales using the maximum likelihood (ML) and Bayesian inference (BI) method based on large single copy (LSC) region. Only the ML tree is shown, because its topology is identical to that of the obtained BI tree. ML supports/BI posterior probabilities values are indicated on the nodes.

**Additional file 9: Figure S2.** Phylogenetic tree reconstruction of Sapindales using the maximum likelihood (ML) and Bayesian inference (BI) method based on inverted repeat (IR) region. Only the ML tree is shown, because its topology is nearly identical to that of the obtained BI tree. ML supports/BI posterior probabilities values are indicated on the nodes. “-” indicates that the node is incongruent between the topology of the ML tree and the Bayesian tree.

**Additional file 10: Figure S3.** Phylogenetic tree reconstruction of Sapindales using the maximum likelihood (ML) and Bayesian inference (BI) method based on small single copy (SSC) region. Only the ML tree is shown, because its topology is identical to that of the obtained BI tree. ML supports/BI posterior probabilities values are indicated on the nodes.

## Data Availability

All data generated or analyzed during this study are included in this published article and the complete chloroplast genome sequences of *Handeliodendron bodinieri* and *Eurycorymbus cavaleriei* are deposited in the genbank with ID no: MK552107 and MK552106, respectively. The accession numbers corresponding to the additional datasets used and analyzed in this study can be found in Table S1. These were retrieved from National Center for Biotechnology Information database.

## Abbreviations

LSC: large single copy
SSC: small single copy
IR: inverted repeat
BI: Bayesian inference
ML: Maximum Likelihood
PCGs: protein-coding genes
rRNA: Ribosomal RNA
SSR: simple sequence repeats
tRNA: Transfer RNA
cp: chloroplast
CTAB: cetyltrimethylammonium bromide
CDS: coding sequence
RSCU: Relative synonymous codon usage
