# Supplementary material for: Comparative genomics and phylogenetic relationships of two endemic and endangered species (Handeliodendron bodinieri and Eurycorymbus cavaleriei) of two monotypic genera within Sapindales
Source: BMC Genomics. 2022 Jan 6;23:27. doi: 10.1186/s12864-021-08259-w (PMC8734052; doi:10.1186/s12864-021-08259-w)
Supplement: Supplementary file 2 — Additional file 2: Table S2. Simple sequence repeats (SSRs) in the Handeliodendron bodinieri chloroplast genome. [file 12864_2021_8259_MOESM2_ESM.docx]

**Table S2 Simple sequence repeats (SSRs) in the *Handeliodendron bodinieri* chloroplast genome**

| **SSR type** | **SSR** | **Start** | **End** | **Location** | **Size** |
| --- | --- | --- | --- | --- | --- |
| p1 | A | 3621 | 3630 | trnK-UUU | 11 |
| p1 | A | 4165 | 4175 | IGS(trnK-UUU-rps16) | 11 |
| p1 | A | 4236 | 4246 | IGS(trnK-UUU-rps16) | 11 |
| p1 | A | 4995 | 5005 | rps16 | 12 |
| p1 | A | 5950 | 5959 | IGS(rps16-trnQ-UUG) | 10 |
| p1 | A | 7144 | 7155 | IGS(psbK-psbI) | 11 |
| p1 | A | 12138 | 12148 | atpF | 10 |
| p1 | A | 12625 | 12634 | IGS(atpF-atpH) | 12 |
| p1 | A | 29974 | 29983 | IGS(psbM-trnD-GUC) | 13 |
| p1 | A | 42750 | 42759 | IGS(psaA-ycf3) | 10 |
| p1 | A | 51720 | 51730 | IGS(ndhC-trnV-UAC) | 14 |
| p1 | A | 55983 | 55993 | IGS(atpB-rbcL) | 12 |
| p1 | A | 59808 | 59819 | IGS(accD-psaI) | 11 |
| p1 | A | 60409 | 60418 | psaI | 10 |
| p1 | A | 64912 | 64921 | psbF | 11 |
| p1 | A | 66133 | 66123 | IGS(psbE-petL） | 10 |
| p1 | A | 70711 | 70720 | IGS(rps12-clpP) | 11 |
| p1 | A | 82053 | 82065 | IGS(rpl14-rpl16) | 10 |
| p1 | A | 108427 | 108436 | IGS(rrn5-trnR-ACG) | 10 |
| p1 | A | 110667 | 110676 | ycf1 | 10 |
| p1 | A | 116867 | 116877 | ndhD | 10 |
| p1 | A | 122391 | 122400 | ndhA | 11 |
| p1 | A | 129385 | 129394 | ycf1 | 11 |
| p1 | A | 131459 | 131469 | IGS(trnN-GUU-trnR-ACG) | 12 |
| p1 | A | 131523 | 131532 | IGS(trnN-GUU-trnR-ACG) | 10 |
| p1 | G | 22603 | 22612 | rpoC1 | 11 |
| p1 | G | 67033 | 67042 | IGS(petG-trnW-CCA) | 10 |
| p1 | T | 3605 | 3618 | trnK-UUU | 10 |
| p1 | T | 7914 | 7923 | IGS(trnS-GCU-trnG-UCC) | 10 |
| p1 | T | 8337 | 8347 | IGS(trnS-GCU-trnG-UCC) | 11 |
| p1 | T | 9457 | 9467 | IGS(trnR-UCU-atpA) | 10 |
| p1 | T | 11835 | 11851 | atpF | 11 |
| p1 | T | 15080 | 15090 | IGS(atpI-rps2) | 10 |
| p1 | T | 18028 | 18145 | rpoC2 | 10 |
| p1 | T | 18135 | 18145 | rpoC2 | 10 |
| p1 | T | 25778 | 25787 | rpoB | 11 |
| p1 | T | 28399 | 28408 | IGS(petN-psbM) | 10 |
| p1 | T | 29033 | 29048 | IGS(psbM-trnD-GUC) | 10 |
| p1 | T | 37055 | 37064 | IGS(trnG-UCC-trnfM-CAU) | 10 |
| p1 | T | 48623 | 48632 | IGS(trnL-UAA-trnF-GAA) | 11 |
| p1 | T | 49595 | 49604 | IGS(trnF-GAA-ndhJ) | 10 |
| p1 | T | 51048 | 51059 | IGS(ndhK-ndhC) | 10 |
| p1 | T | 51062 | 51072 | IGS(ndhK-ndhC) | 10 |
| p1 | T | 52469 | 52479 | trnV-UAC | 11 |
| p1 | T | 55390 | 55399 | atpB | 11 |
| p1 | T | 55872 | 55881 | IGS(atpB-rbcL) | 10 |
| p1 | T | 60455 | 60464 | IGS(psaI-ycf4) | 10 |
| p1 | T | 61594 | 61603 | IGS(ycf4-cemA) | 10 |
| p1 | T | 51651 | 61660 | IGS(ycf4-cemA) | 10 |
| p1 | T | 65747 | 65756 | IGS(psbE-petL） | 12 |
| p1 | T | 65964 | 65974 | IGS(psbE-petL） | 13 |
| p1 | T | 66005 | 66016 | IGS(psbE-petL） | 10 |
| p1 | T | 67234 | 67246 | IGS(trnW-CCA-trnP-UGG) | 11 |
| p1 | T | 68020 | 68032 | IGS(psaJ-rpl33) | 10 |
| p1 | T | 68752 | 68761 | IGS(rpl33-rps18) | 13 |
| p1 | T | 70467 | 70477 | IGS(rpl20-rps12) | 10 |
| p1 | T | 71247 | 71256 | clpP | 11 |
| p1 | T | 71341 | 71350 | clpP | 17 |
| p1 | T | 74935 | 74944 | IGS(psbB-psbT) | 11 |
| p1 | T | 75713 | 75723 | IGS(psbH-petB) | 10 |
| p1 | T | 81011 | 81021 | IGS(infA-rps8) | 10 |
| p1 | T | 81520 | 81530 | IGS(rps8-rpl15) | 16 |
| p1 | T | 108832 | 108841 | IGS(trnR-ACG-trnN-GUU) | 11 |
| p1 | T | 108895 | 108905 | IGS(trnR-ACG-trnN-GUU) | 10 |
| p1 | T | 114422 | 114440 | IGS(rpl32-trnL-UAG) | 10 |
| p1 | T | 116578 | 116588 | IGS(ccsA-ndhD) | 11 |
| p1 | T | 119888 | 119897 | IGS(ndhG-ndhI) | 11 |
| p1 | T | 122423 | 122432 | ndhA | 11 |
| p1 | T | 124309 | 124319 | IGS(ndhH-rps15) | 19 |
| p1 | T | 124549 | 124559 | rps15 | 11 |
| p1 | T | 125331 | 125341 | ycf1 | 10 |
| p1 | T | 127756 | 127765 | ycf1 | 11 |
| p1 | T | 127877 | 127888 | ycf1 | 11 |
| p1 | T | 129688 | 129697 | ycf1 | 10 |
| p1 | T | 131928 | 131937 | IGS(trnR-ACG-rrn5) | 10 |
| p2 | AT | 117984 | 117993 | ndhD | 10 |
| p2 | TA | 78115 | 78126 | petD | 12 |
| p2 | TA | 67246 | 67255 | IGS(trnW-CCA-trnP-UGG) | 10 |
| p2 | TA | 19507 | 19516 | rpoC2 | 10 |
| p2 | TA | 46884 | 46893 | IGS(rps4-trnT-UGU) | 10 |
| p2 | TA | 78115 | 78126 | petD | 12 |
| p2 | TC | 61895 | 61904 | cemA | 10 |
| p3 | AAG | 94568 | 94579 | IGS(ycf2-trnL-CAA) | 12 |
| p3 | AAT | 113027 | 113038 | IGS(ndhF-rpl32) | 12 |
| p3 | ATA | 49635 | 49646 | IGS(trnF-GAA-ndhJ) | 12 |
| p3 | ATA | 55447 | 55455 | IGS(atpB-rbcL) | 12 |
| p3 | CTT | 145785 | 145796 | IGS(trnL-CAA-ycf2) | 12 |
| p4 | ATAC | 49096 | 49107 | IGS(trnF-GAA-ndhJ) | 12 |
| p4 | ATAG | 31570 | 31581 | IGS(trnE-UUC-trnT-GGU) | 12 |
| p4 | CAAA | 29419 | 29430 | IGS(psbM-trnD-GUC) | 12 |
| p4 | TAAT | 36649 | 36660 | IGS(psbZ-trnG-GCC) | 12 |
| p4 | TAGA | 68038 | 68049 | IGS(psaJ-rpl33) | 12 |
| p4 | TAGT | 59780 | 59791 | IGS(accD-psaI) | 12 |
| p4 | TCTA | 32182 | 32193 | IGS(trnT-GGU-psbD) | 12 |
| p4 | TTAT | 9626 | 9637 | IGS(trnR-UCU-atpA) | 12 |
| p4 | TTTA | 8069 | 8080 | IGS(trns-GCU-trnG-UCC) | 12 |
| p5 | TTCTA | 12174 | 12188 | atpF | 15 |
