# Supplementary material for: Comparative genomics and phylogenetic relationships of two endemic and endangered species (Handeliodendron bodinieri and Eurycorymbus cavaleriei) of two monotypic genera within Sapindales
Source: BMC Genomics. 2022 Jan 6;23:27. doi: 10.1186/s12864-021-08259-w (PMC8734052; doi:10.1186/s12864-021-08259-w)
Supplement: Supplementary file 3 — Additional file 3: Table S3. Simple sequence repeats (SSRs) in Eurycorymbus cavaleriei chloroplast genome. [file 12864_2021_8259_MOESM3_ESM.docx]

**Table S3 Simple sequence repeats (SSRs) in *Eurycorymbus cavaleriei* chloroplast genome**

| **SSR type** | **SSR** | **Start** | **End** | **Location** | **Size** |
| --- | --- | --- | --- | --- | --- |
| p1 | A | 3789 | 3798 | trnK-UUU | 10 |
| p1 | A | 4877 | 4886 | IGS(trnK-UUU-rps16) | 10 |
| p1 | A | 7055 | 7064 | IGS(rps16-trnQ-UUG) | 10 |
| p1 | A | 9514 | 9523 | IGS(trnS-GCU-trnG-UCC) | 10 |
| p1 | A | 31753 | 31762 | IGS(psbM-trnD-GUC) | 10 |
| p1 | A | 61918 | 61927 | IGS(accD-psaI) | 10 |
| p1 | A | 62260 | 62269 | psaI | 10 |
| p1 | A | 63888 | 63897 | IGS(ycf4-cemA) | 10 |
| p1 | A | 66993 | 67002 | psbF | 10 |
| p1 | A | 68267 | 68276 | IGS(psbE-petL) | 10 |
| p1 | A | 75350 | 75361 | IGS(clpP-psbB) | 12 |
| p1 | A | 80955 | 80965 | IGS(petD-rpoA) | 11 |
| p1 | A | 113521 | 113533 | ycf1 | 13 |
| p1 | A | 116522 | 116536 | IGS(ndhF-rpl32) | 15 |
| p1 | A | 117404 | 117413 | IGS(rpl32-trnL-UAG) | 10 |
| p1 | A | 127530 | 127539 | IGS(rps15-ycf1) | 10 |
| p1 | A | 129840 | 129849 | ycf1 | 10 |
| p1 | A | 131633 | 131645 | ycf1 | 13 |
| p1 | G | 36699 | 36709 | psb | 11 |
| p1 | T | 5902 | 5911 | rps16 | 10 |
| p1 | T | 9153 | 9162 | IGS(psbI-trnS-GCU) | 12 |
| P1 | T | 9750 | 9759 | IGS(trnS-GCU-trnG-UCC) | 10 |
| p1 | T | 11337 | 11346 | IGS(trnR-UCU-atpA) | 12 |
| p1 | T | 14342 | 14355 | IGS(atpF-atpH) | 14 |
| p1 | T | 19663 | 19672 | rpoC2 | 10 |
| p1 | T | 19770 | 19780 | rpoC2 | 11 |
| p1 | T | 27442 | 27451 | rpoB | 10 |
| p1 | T | 30983 | 30993 | IGS(psbM-trnD-GUC) | 11 |
| p1 | T | 52754 | 52763 | IGS(ndhK-ndhC) | 10 |
| p1 | T | 57128 | 57137 | atpB | 10 |
| p1 | T | 61597 | 61606 | IGS(accD-psaI) | 10 |
| p1 | T | 68254 | 68265 | IGS(psbE-petL) | 12 |
| p1 | T | 72853 | 72868 | IGS(rps12-clpP) | 16 |
| p1 | T | 73419 | 73428 | clpP | 10 |
| p1 | T | 77847 | 77856 | IGS(psbH-petB) | 10 |
| p1 | T | 127235 | 127245 | rps15 | 11 |
| p1 | T | 129665 | 129674 | ycf1 | 10 |
| p1 | T | 132032 | 132044 | ycf1 | 13 |
| p2 | AT | 11185 | 11196 | IGS(trnR-UCU-atpA） | 12 |
| p2 | AT | 49620 | 49629 | IGS(trnG-UGU-trnL-UAA) | 10 |
| p2 | AT | 124833 | 124844 | ndhA | 12 |
| p2 | TA | 21142 | 21151 | rpoC2 | 10 |
| p2 | TA | 69343 | 69360 | IGS(trnW-CCA-trnP-UGG) | 18 |
| p3 | AAG | 96840 | 96851 | IGS(ycf2-trnL-CAA) | 12 |
| p3 | ATA | 350 | 361 | IGS(trnH-GUG-psbA) | 12 |
| p3 | ATA | 38683 | 38697 | IGS(psbZ-trnG-UCC) | 15 |
| p3 | ATA | 51330 | 51341 | IGS(trnF-GAA-ndhJ) | 12 |
| p3 | ATT | 113824 | 113838 | ndhF | 15 |
| p3 | CTT | 148714 | 148725 | IGS(trnL-ycf2) | 12 |
| p3 | TAA | 45366 | 45377 | ycf3 | 12 |
| p3 | TTA | 15372 | 15383 | IGS(atpH-atpI) | 12 |
| p3 | TTC | 18100 | 18111 | rpoC2 | 12 |
| P4 | AATA | 6717 | 6728 | IGS(rps16-trnQ-UUG) | 12 |
| P4 | ATAA | 6738 | 6749 | IGS(rps17-trnQ-UUG) | 12 |
| p4 | ATAG | 33469 | 33480 | IGS(trnE-UUC-trnT-GGU) | 12 |
| p4 | GATA | 7371 | 7382 | IGS(rps17-trnQ-UUG) | 10 |
| p4 | TAGA | 34278 | 34289 | IGS(trnT-GGU-psbD) | 12 |
| p4 | TCAA | 5029 | 5040 | IGS(trnK-UUU-rps16) | 12 |
| p4 | TCTA | 34261 | 34272 | IGS(trnT-GGU-psbD) | 12 |
| P4 | TTTA | 9757 | 9768 | IGS(trnS-GCU-trnG-UCC) | 12 |
