# Supplementary material for: Comparative genomics and phylogenetic relationships of two endemic and endangered species (Handeliodendron bodinieri and Eurycorymbus cavaleriei) of two monotypic genera within Sapindales
Source: BMC Genomics. 2022 Jan 6;23:27. doi: 10.1186/s12864-021-08259-w (PMC8734052; doi:10.1186/s12864-021-08259-w)
Supplement: Supplementary file 4 — Additional file 4: Table S4. Relative synonymous codon usage (RSCU) of Handeliodendron bodinieri chloroplast genome. [file 12864_2021_8259_MOESM4_ESM.docx]

**Table S4 Relative synonymous codon usage (RSCU) of *Handeliodendron bodinieri* chloroplast genome**

| Amino acids | Codon | RSCU | No. | Amino acid frequencies |
| --- | --- | --- | --- | --- |
| Ala | GCU | 1.77 | 601 | 5.2% |
|  | GCC | 0.65 | 220 |  |
|  | GCA | 1.09 | 370 |  |
|  | GCG | 0.49 | 165 |  |
| Arg | CGU | 1.24 | 322 | 6.0% |
|  | CGC | 0.46 | 120 |  |
|  | CGA | 1.36 | 354 |  |
|  | CGG | 0.51 | 133 |  |
|  | AGA | 1.8 | 469 |  |
|  | AGG | 0.63 | 163 |  |
| Asn | AAU | 1.54 | 973 | 4.9% |
|  | AAC | 0.46 | 294 |  |
| Asp | GAU | 1.59 | 831 | 4.0% |
|  | GAC | 0.41 | 215 |  |
| Cys | UGU | 1.45 | 216 | 1.1% |
|  | UGC | 0.55 | 81 |  |
| Gln | CAA | 1.53 | 709 | 3.6% |
|  | CAG | 0.47 | 217 |  |
| Glu | GAA | 1.49 | 987 | 5.1% |
|  | GAG | 0.51 | 341 |  |
| Gly | GGU | 1.29 | 580 | 6.9% |
|  | GGC | 0.38 | 172 |  |
|  | GGA | 1.63 | 732 |  |
|  | GGG | 0.7 | 313 |  |
| His | CAU | 1.49 | 478 | 2.5% |
|  | CAC | 0.51 | 163 |  |
| Ile | AUU | 1.48 | 1091 | 8.5% |
|  | AUC | 0.6 | 446 |  |
|  | AUA | 0.92 | 678 |  |
| Leu | UUA | 1.78 | 810 | 10.5% |
|  | UUG | 1.23 | 559 |  |
|  | CUU | 1.27 | 580 |  |
|  | CUC | 0.43 | 195 |  |
|  | CUA | 0.84 | 381 |  |
|  | CUG | 0.46 | 210 |  |
| Lys | AAA | 1.49 | 1044 | 5.4% |
|  | AAG | 0.51 | 362 |  |
| Met | AUG | 1 | 597 | 2.3% |
| Phe | UUU | 1.29 | 996 | 5.9% |
|  | UUC | 0.71 | 544 |  |
| Pro | CCU | 1.54 | 413 | 4.1% |
|  | CCC | 0.8 | 214 |  |
|  | CCA | 1.16 | 311 |  |
|  | CCG | 0.51 | 137 |  |
| Ser | UCU | 1.59 | 539 | 7.8% |
|  | UCC | 1 | 337 |  |
|  | UCA | 1.25 | 423 |  |
|  | UCG | 0.6 | 202 |  |
|  | AGU | 1.17 | 397 |  |
|  | AGC | 0.4 | 134 |  |
| Thr | ACU | 1.57 | 508 | 5.0% |
|  | ACC | 0.78 | 253 |  |
|  | ACA | 1.19 | 386 |  |
|  | ACG | 0.46 | 150 |  |
| Trp | UGG | 1 | 455 | 1.7% |
| Tyr | UAU | 1.63 | 777 | 3.7% |
|  | UAC | 0.37 | 175 |  |
| Val | GUU | 1.45 | 516 | 5.5% |
|  | GUC | 0.51 | 182 |  |
|  | GUA | 1.45 | 515 |  |
|  | GUG | 0.59 | 208 |  |
| TER | UAA | 1.71 | 48 | 0.3% |
|  | UAG | 0.71 | 20 |  |
|  | UGA | 0.57 | 16 |  |
