# Supplementary material for: Comparative genomics and phylogenetic relationships of two endemic and endangered species (Handeliodendron bodinieri and Eurycorymbus cavaleriei) of two monotypic genera within Sapindales
Source: BMC Genomics. 2022 Jan 6;23:27. doi: 10.1186/s12864-021-08259-w (PMC8734052; doi:10.1186/s12864-021-08259-w)
Supplement: Supplementary file 5 — Additional file 5: Table S5. Relative synonymous codon usage (RSCU) of Eurycorymbus cavaleriei chloroplast genome. [file 12864_2021_8259_MOESM5_ESM.docx]

**Table S5 Relative synonymous codon usage (RSCU) of *Eurycorymbus cavaleriei* chloroplast genome**

| Amino acids | Codon | RSCU | No. | Amino acid frequencies |
| --- | --- | --- | --- | --- |
| Ala | GCU | 1.76 | 623 | 5.4% |
|  | GCC | 0.64 | 225 |  |
|  | GCA | 1.07 | 380 |  |
|  | GCG | 0.53 | 188 |  |
| Arg | CGU | 1.23 | 333 | 6.2% |
|  | CGC | 0.48 | 131 |  |
|  | CGA | 1.34 | 363 |  |
|  | CGG | 0.47 | 128 |  |
|  | AGA | 1.8 | 488 |  |
|  | AGG | 0.69 | 186 |  |
| Asn | AAU | 1.51 | 972 | 4.9% |
|  | AAC | 0.49 | 318 |  |
| Asp | GAU | 1.57 | 817 | 3.9% |
|  | GAC | 0.43 | 227 |  |
| Cys | UGU | 1.43 | 214 | 1.1% |
|  | UGC | 0.57 | 85 |  |
| Gln | CAA | 1.51 | 710 | 3.5% |
|  | CAG | 0.49 | 229 |  |
| Glu | GAA | 1.46 | 998 | 5.2% |
|  | GAG | 0.54 | 367 |  |
| Gly | GGU | 1.24 | 561 | 6.9% |
|  | GGC | 0.43 | 194 |  |
|  | GGA | 1.59 | 719 |  |
|  | GGG | 0.75 | 339 |  |
| His | CAU | 1.48 | 477 | 2.4% |
|  | CAC | 0.52 | 168 |  |
| Ile | AUU | 1.46 | 1087 | 8.4% |
|  | AUC | 0.61 | 457 |  |
|  | AUA | 0.92 | 687 |  |
| Leu | UUA | 1.79 | 832 | 10.6% |
|  | UUG | 1.18 | 551 |  |
|  | CUU | 1.28 | 594 |  |
|  | CUC | 0.47 | 221 |  |
|  | CUA | 0.85 | 397 |  |
|  | CUG | 0.42 | 197 |  |
| Lys | AAA | 1.47 | 1054 | 5.4% |
|  | AAG | 0.53 | 381 |  |
| Met | AUG | 1 | 614 | 2.3% |
| Phe | UUU | 1.3 | 996 | 5.8% |
|  | UUC | 0.7 | 540 |  |
| Pro | CCU | 1.52 | 414 | 4.1% |
|  | CCC | 0.84 | 230 |  |
|  | CCA | 1.12 | 307 |  |
|  | CCG | 0.52 | 142 |  |
| Ser | UCU | 1.65 | 559 | 7.7% |
|  | UCC | 1.06 | 359 |  |
|  | UCA | 1.22 | 414 |  |
|  | UCG | 0.56 | 190 |  |
|  | AGU | 1.13 | 382 |  |
|  | AGC | 0.38 | 128 |  |
| Thr | ACU | 1.52 | 503 | 5.0% |
|  | ACC | 0.79 | 262 |  |
|  | ACA | 1.21 | 400 |  |
|  | ACG | 0.47 | 156 |  |
| Trp | UGG | 1 | 458 | 1.7% |
| Tyr | UAU | 1.61 | 775 | 3.6% |
|  | UAC | 0.39 | 185 |  |
| Val | GUU | 1.47 | 534 | 5.5% |
|  | GUC | 0.51 | 186 |  |
|  | GUA | 1.47 | 534 |  |
|  | GUG | 0.55 | 202 |  |
| TER | UAA | 1.62 | 47 | 0.3% |
|  | UAG | 0.76 | 22 |  |
|  | UGA | 0.62 | 18 |  |
