# Supplementary material for: Comparative genomics and phylogenetic relationships of two endemic and endangered species (Handeliodendron bodinieri and Eurycorymbus cavaleriei) of two monotypic genera within Sapindales
Source: BMC Genomics. 2022 Jan 6;23:27. doi: 10.1186/s12864-021-08259-w (PMC8734052; doi:10.1186/s12864-021-08259-w)
Supplement: Supplementary file 6 — Additional file 6: Table S6. Positive selected sites detected in the cp genome of the Dodonaeoideae + Sapindoideae. Table S7. Positive selected sites detected in the cp genome of the subfamily Hippocastanoideae. [file 12864_2021_8259_MOESM6_ESM.docx]

**Table S6 Positive selected sites detected in the cp genome of the Dodonaeoideae + Sapindoideae.**

| M8 | Gene Name | Region | Selected Sites | Pr (w > 1) | Number of Selected Sites |
| --- | --- | --- | --- | --- | --- |
| NEB and BEB | clpP | LSC | 2908 A | 0.971* | 1 |
|  | ndhF | SSC | 5438 T, 5655 Y, 5656 Y | 0.972*, 1.000**, 0.973* | 3 |
|  | petA | LSC | 7265 P | 0.979* | 1 |
|  | rpoC1 | LSC | 14863 W | 0.977* | 1 |
|  | rpoC2 | LSC | 16595 K, 16597 K | 0.961*, 0.995** | 2 |
|  | rps11 | LSC | 16810 A | 0.951* | 1 |

*: p < 0.05; **: p < 0.01

**Table S7 Positive selected sites detected in the cp genome of the subfamily Hippocastanoideae.**

| M8 | Gene Name | Region | Selected Sites | Pr (w > 1) | Number of Selected Sites |
| --- | --- | --- | --- | --- | --- |
| NEB | atpA | LSC | 935 A | 0.986* | 1 |
|  | ndhF | SSC | 5570 S, 5716 S, 5717 N | 0.977*, 1.000**, 1.000** | 3 |
|  | rpl23 | IR | 12793 Y, 12794 D | 0.991**, 0.988* | 2 |
|  | rpoC2 | LSC | 16346 E - 16422 Y | 0.960* ~ 1.000** | 23 |
|  | ycf1 | SSC | 18066 Q - 19464 L | 0.952* ~ 1.000** | 10 |
| BEB | atpA | LSC | 935 A | 0.986* | 1 |
|  | ndhD | SSC | 4780 F | 0.972* | 1 |
|  | ndhF | SSC | 5570 S, 5716 S, 5717 | 0.977*, 1.000** | 3 |
|  | rpl23 | IR | 12793 Y, 12794 D | 0.988*, 0.989* | 2 |
|  | rpoC2 | LSC | 16346 E - 16421 W | 0.954* ~ 1.000** | 32 |
|  | ycf1 | SSC | 18066 Q - 19464 L | 0.967* ~ 1.000** | 10 |

*: p < 0.05; **: p < 0.01
