# Supplementary material for: Comparative genomics and phylogenetic relationships of two endemic and endangered species (Handeliodendron bodinieri and Eurycorymbus cavaleriei) of two monotypic genera within Sapindales
Source: BMC Genomics. 2022 Jan 6;23:27. doi: 10.1186/s12864-021-08259-w (PMC8734052; doi:10.1186/s12864-021-08259-w)
Supplement: Supplementary file 7 — Additional file 7: Table S8. Best-fit Models in ML and BI analysis. [file 12864_2021_8259_MOESM7_ESM.docx]

Table S8 Best-fit models in ML and BI analysis phylogenetic analyses with different datasets

| Datasets | Model in BI | Model in ML |
| --- | --- | --- |
| IR | GTR+F+I+G4 | K3Pu+F+R3 |
| LSC | GTR+F+I+G4 | GTR+F+R4 |
| SSC | GTR+F+I+G4 | TVM+F+R4 |
| Whole cp genome | GTR+F+I+G4 | GTR+F+R4 |
| CDS | GTR+F+I+G4 | TVM+F+R3 |
