# Supplementary figures and images for: Comparative genomics and phylogenetic relationships of two endemic and endangered species (Handeliodendron bodinieri and Eurycorymbus cavaleriei) of two monotypic genera within Sapindales
Source: BMC Genomics. 2022 Jan 6;23:27. doi: 10.1186/s12864-021-08259-w (PMC8734052; doi:10.1186/s12864-021-08259-w)

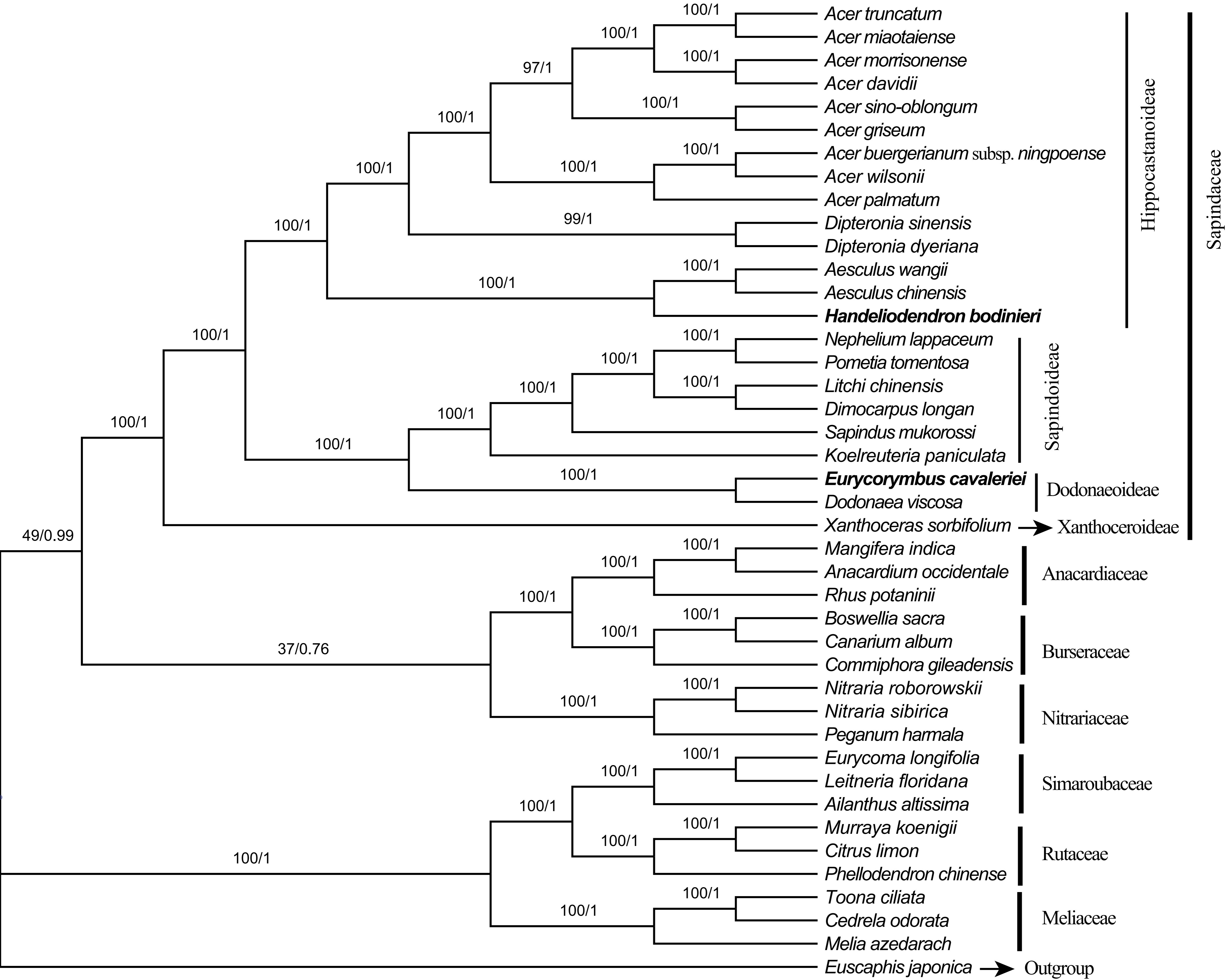

Supplement: Supplementary file 8 — Additional file 8: Figure S1. Phylogenetic tree reconstruction of Sapindales using the maximum likelihood (ML) and Bayesian inference (BI) method based on large single copy (LSC) region. Only the ML tree is shown, because its topology is identical to that of the obtained BI tree. ML supports/BI posterior probabilities values are indicated on the nodes. [file 12864_2021_8259_MOESM8_ESM.pdf]

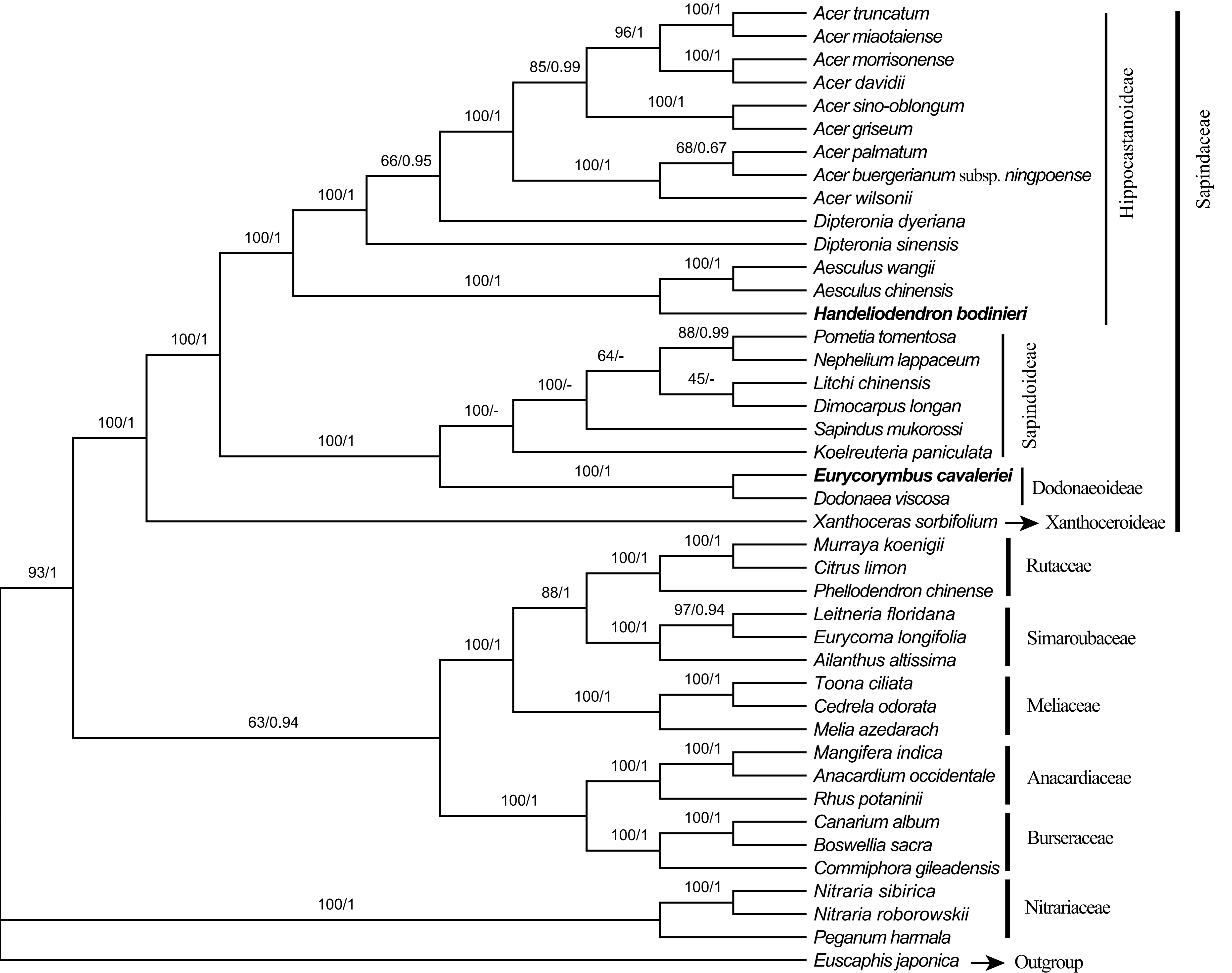

Supplement: Supplementary file 9 — Additional file 9: Figure S2. Phylogenetic tree reconstruction of Sapindales using the maximum likelihood (ML) and Bayesian inference (BI) method based on inverted repeat (IR) region. Only the ML tree is shown, because its topology is nearly identical to that of the obtained BI tree. ML supports/BI posterior probabilities values are indicated on the nodes. “-” indicates that the node is incongruent between the topology of the ML tree and the Bayesian tree. [file 12864_2021_8259_MOESM9_ESM.pdf]

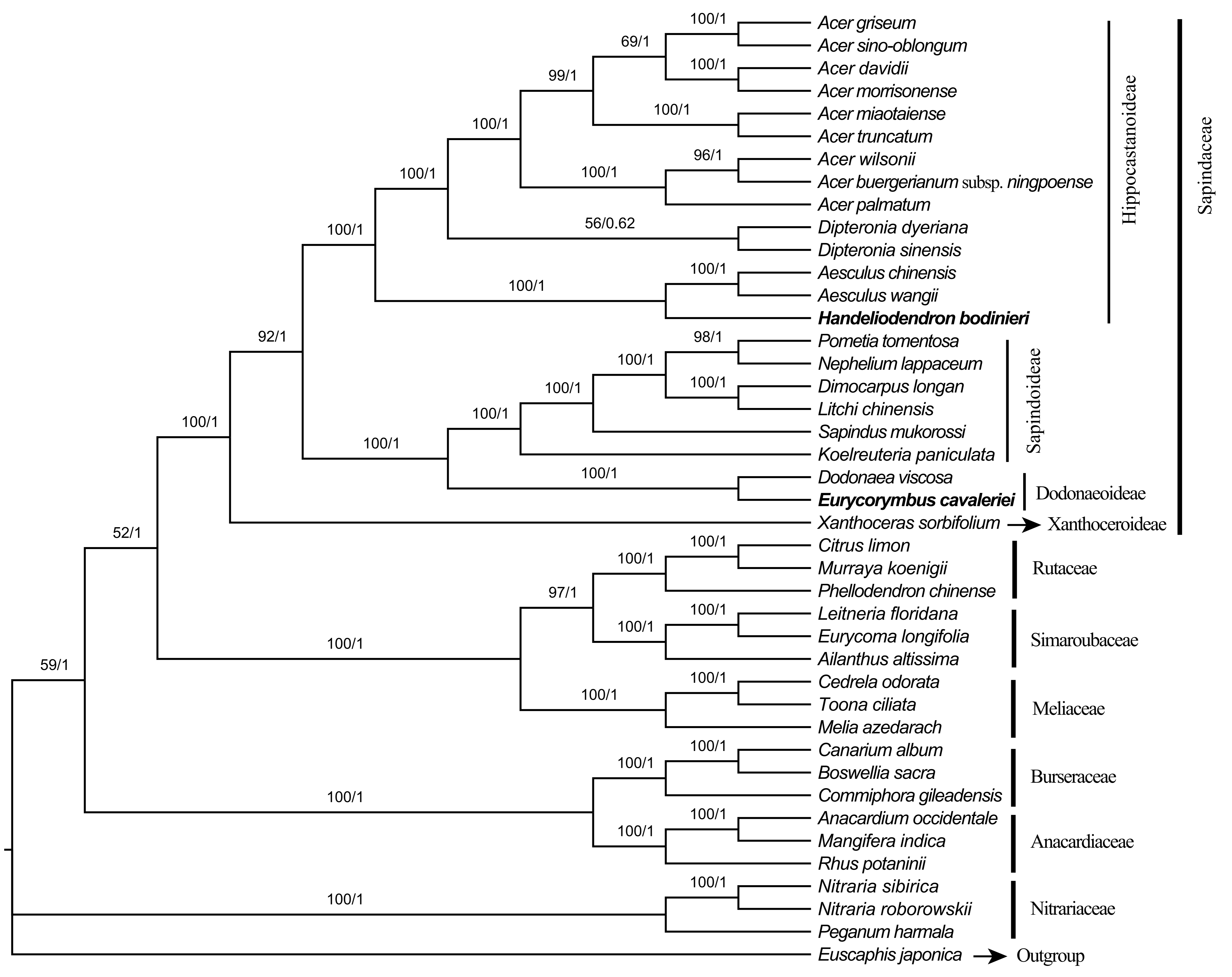

Supplement: Supplementary file 10 — Additional file 10: Figure S3. Phylogenetic tree reconstruction of Sapindales using the maximum likelihood (ML) and Bayesian inference (BI) method based on small single copy (SSC) region. Only the ML tree is shown, because its topology is identical to that of the obtained BI tree. ML supports/BI posterior probabilities values are indicated on the nodes. [file 12864_2021_8259_MOESM10_ESM.pdf]
